# DisoFLAG: accurate prediction of protein intrinsic disorder and its functions using graph-based interaction protein language model

**DOI:** 10.1186/s12915-023-01803-y

**Published:** 2024-01-02

**Authors:** Yihe Pang, Bin Liu

**Affiliations:** 1 School of Computer Science and Technology, Beijing Institute of Technology, No. 5, South Zhongguancun Street, Beijing, Haidian District 100081 China; 2 Advanced Research Institute of Multidisciplinary Science, Beijing Institute of Technology, No. 5, South Zhongguancun Street, Beijing, Haidian District 100081 China

**Keywords:** Protein intrinsic disorder, Disordered function prediction, Protein language model, Graph-based interaction protein language model

## Abstract

Intrinsically disordered proteins and regions (IDPs/IDRs) are functionally important proteins and regions that exist as highly dynamic conformations under natural physiological conditions. IDPs/IDRs exhibit a broad range of molecular functions, and their functions involve binding interactions with partners and remaining native structural flexibility. The rapid increase in the number of proteins in sequence databases and the diversity of disordered functions challenge existing computational methods for predicting protein intrinsic disorder and disordered functions. A disordered region interacts with different partners to perform multiple functions, and these disordered functions exhibit different dependencies and correlations. In this study, we introduce DisoFLAG, a computational method that leverages a graph-based interaction protein language model (GiPLM) for jointly predicting disorder and its multiple potential functions. GiPLM integrates protein semantic information based on pre-trained protein language models into graph-based interaction units to enhance the correlation of the semantic representation of multiple disordered functions. The DisoFLAG predictor takes amino acid sequences as the only inputs and provides predictions of intrinsic disorder and six disordered functions for proteins, including protein-binding, DNA-binding, RNA-binding, ion-binding, lipid-binding, and flexible linker. We evaluated the predictive performance of DisoFLAG following the Critical Assessment of protein Intrinsic Disorder (CAID) experiments, and the results demonstrated that DisoFLAG offers accurate and comprehensive predictions of disordered functions, extending the current coverage of computationally predicted disordered function categories. The standalone package and web server of DisoFLAG have been established to provide accurate prediction tools for intrinsic disorders and their associated functions.

**Supplementary Information:**

The online version contains supplementary material available at 10.1186/s12915-023-01803-y.

## Background

Proteins are essential macromolecules in living organisms, and the majority of proteins fold into specific and ordered three-dimensional conformations to perform their functions. Intrinsically disordered proteins and regions (IDPs/IDRs) are a special class of proteins or regions that exist without stable fold structures under native physiologic conditions. Despite lacking well-defined tertiary structures, IDPs/IDRs play essential roles in a wide range of biological processes, such as cell signaling [1], DNA regulation [2], and post-translational modification [3]. IDP/IDRs are also associated with many human diseases [4], including neurodegenerative disease [5, 6], diabetes [7], cancer [1, 8], and cardiovascular disease [9, 10]. The flexibility of IDRs in their structures enables them to bind many molecular ligands, thus making them effective drug targets [11]. Therefore, identifying disordered regions in proteins and understanding their functional roles will contribute to rational drug design and improve the efficiency of new drug development [12, 13].

Experimental characterization of IDPs/IDRs in the wet lab is expensive and labor-intensive. With the massive growth in the number of protein sequences available in databases [14], computationally predicting IDP/IDRs directly from sequences is considered a feasible approach. Numerous computational methods by leveraging different sequence features and computing techniques have been developed for identifying IDRs in proteins, such as SPOT-disorder [15], DISOPRED3 [16], SPINE-D [17], AUCpreD [18], IDP-Seq2seq [19], SPOT-Disorder2 [20], and fIDPnn. Their predictive qualities were comprehensively evaluated by a community-driven Critical Assessment of protein Intrinsic Disorder (CAID) [21]. The first edition of CAID (CAID1) [21] evaluated a total of 32 disorder predictors, and the second round of CAID (CAID2) [22, 23] was recently completed and involved the evaluations of a total of 46 different computational methods.

IDP/IDRs perform multiple critical functions in living organisms [24]. These functions can be broadly classified into two categories: the binding functions that arise from interacting with partners and the non-binding functions that originate from their native structural flexibility [24, 25]. Many computational predictive methods have been developed focusing on binding regions in IDRs, including methods for identifying protein-binding sites [26–31], DNA-binding sites [26, 29, 30], RNA-binding sites [26, 29, 30], and lipid-binding sites [32]. There are several predictors [33–35] available for identifying the molecular recognition features (MoRFs) within IDRs, which are disordered regions that bind to target protein domains in a process known as disorder-to-order transition. Linker serves as the primary function of the non-binding category, playing a critical role in linking multiple structured domains and permitting domain movements between catalytic sites [36, 37]. Methods [38–40] for identifying disordered flexible linkers (DFLs) from protein sequences have been developed. Besides, a single IDR in proteins is able to bind with different ligands to perform multiple functions, and several prediction tools such as DisoRDPbind [29] and DeepDISOBind [30] have been designed to provide predictions for multiple types of disordered binding regions, including IDRs involved in protein binding, DNA binding, and RNA binding. fIDPnn [26] is an available method for predicting both the binding and non-binding functions of IDRs. Due to the previous efforts in disorder functional prediction, the CAID1 has included the assessment of disordered binding regions [21], and the recent CAID2 has extended the evaluation to the prediction of disordered linkers [22, 23]. As the results indicated by CAID, there still exists substantial room for improvement in the current predictors. (1) Insufficient coverage of functional predictions: IDRs perform multiple functions, and predictors covering more functional categories are required. (2) The multiple functions of intrinsically disordered proteins/regions are dependent and interrelated; the current methods do not take into account the functional correlations, leading to low predictive accuracy.

The biological sequences and natural languages share three hierarchical levels of similarities. (1) Genetic similarity: the language ability in biological organisms, including humans, is involved in specific genes [41]. Both the origin of language and the evolution of biological species stem from genetic inheritance and variation. (2) Evolutionary similarity: biological organisms and natural languages share similar mechanisms of evolution. Natural language is an exclusive characteristic of human beings, and both the development of language and the evolution of species are directed by natural selection [42]. (3) Formal similarity: biological sequences exhibit similar arrangement rules and combination patterns to those observed in natural languages [43], for example, the frequency of occurrence of words in language and domains in proteome following the same form of Zipf’s law [44]. These similarities fundamentally ensure the efficacy of applying natural language processing (NLP) techniques in the analysis of biological sequences [45–47]. The protein language model (PLM) stands out as one of the most representative approaches [48–50]. Its capability to capture semantic information of protein sequence, structure, and function [51] has demonstrated significant potential in a series of studies, including protein design [52–54] and protein function prediction [55]. In this study, we investigated how to incorporate the protein semantic knowledge to facilitate computational predictions of disordered regions and their functions.

**Fig. 1:**
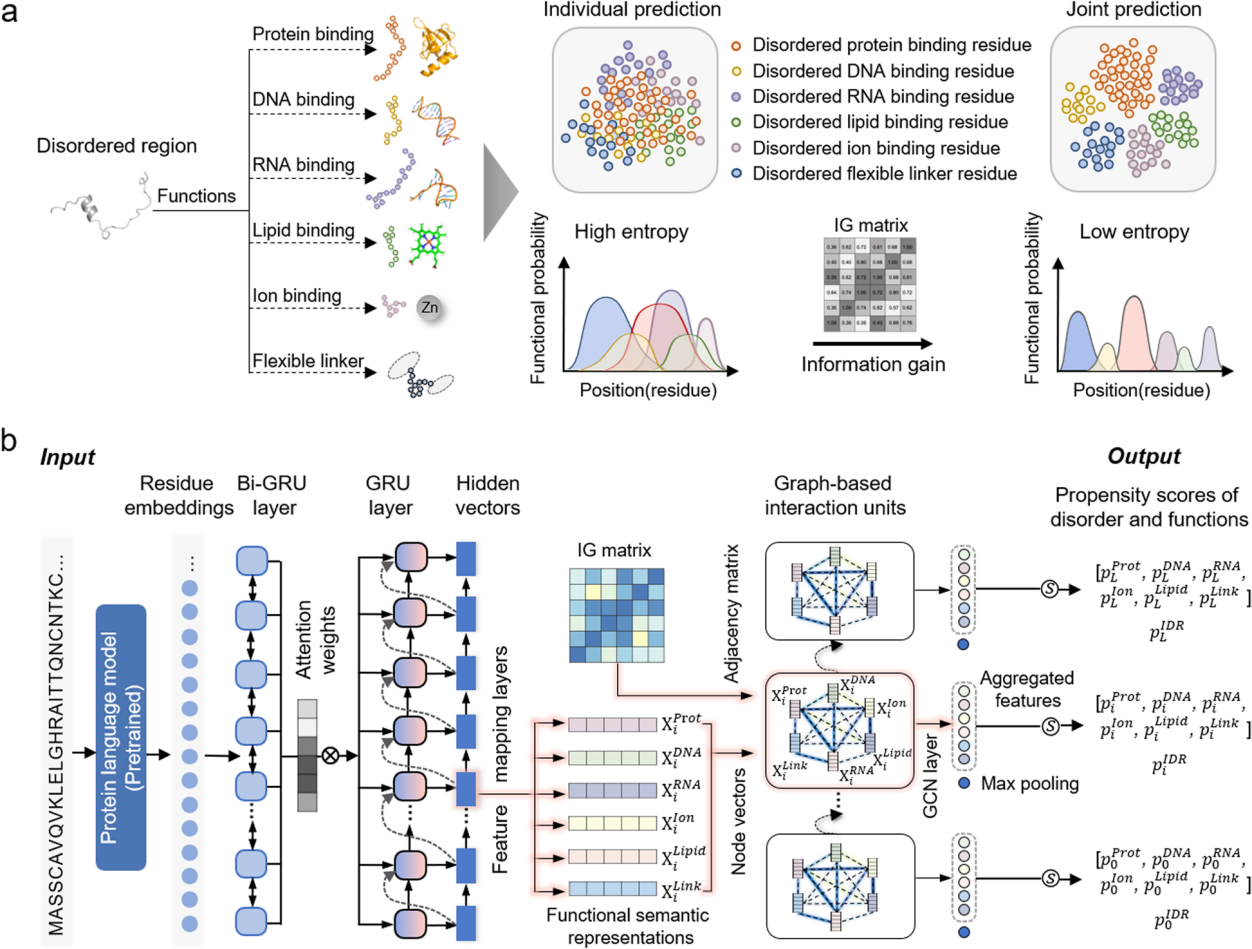
Schematic overview of DisoFLAG. **a** DisoFLAG provides predictions of six functions for intrinsically disordered regions in proteins. Joint prediction of the six functional regions results in a lower information entropy compared to individual prediction. The reduction in information entropy is known as information gain (IG), which reflects the correlation between different functions. High IG, strong correlation. **b** The graph-based interaction protein language model (GiPLM) architecture employed in DisoFLAG. The bi-directional gated recurrent unit (Bi-GRU) layer is used to capture the protein contextual semantic information based on the residue embeddings extracted from the pre-trained protein language model. The subsequent attention-based gated recurrent unit (GRU) layer is used to model the global correlation among sequences and produces a hidden representation for each residue. The feature mapping layers are used to generate six different function embedding vectors (X_*i*_) for each residue. Subsequently, for each residue, the graph-based interaction unit models six functions and their correlations as a functional graph, utilizing function embedding vectors (X_*i*_) as node representations and pre-calculated IG matrix as the weighted adjacency matrix for graph edges. Finally, the propensity scores for disorder and six disordered functions were calculated based on the semantic correlation features aggregated on the functional graph by the graph convolutional network (GCN) layer

Here, we describe a computational method for jointly predicting disorder and multiple disordered functions, termed DisoFLAG. The DisoFLAG employs a graph-based interaction protein language model (GiPLM) to provide six functional predictions for the intrinsic disorder, including protein-binding, DNA-binding, RNA-binding, ion-binding, lipid-binding, and flexible linker (see Fig. 1a). The GiPLM integrates the protein semantic information obtained from pre-trained protein language model into graph-based interaction units. The graph-based interaction unit models the multiple disordered functions as a graph to learn the semantic correlation features among different disordered functions. Then, the propensity scores for disorder and six functions were calculated based on the semantic correlation features aggregated by the graph convolutional network (GCN) layer (see Fig. 1b). Following the CAID, we performed evaluations of DisoFLAG on the CAID2 dataset and two independent test datasets built from the latest DisProt database. The evaluation results demonstrated that DisoFLAG achieves relatively higher performance in predicting disorder and disordered functions. We provide a standalone package and a convenient web server for DisoFLAG.

## Methods

### Benchmark dataset of disorder functions

The DisProt [56–58] database provided the functional annotations of intrinsically disordered protein/region (IDP/IDR) following the Intrinsically Disordered Proteins Ontology (IDPO) and the Gene Ontology (GO) schemas. We investigated all the ontology terms in DisProt and obtained functional annotation term collections for protein-, DNA-, RNA-, ion-, and lipid-binding and flexible linker (Additional file 1: Table S1). Following previous studies [21, 29, 59], we annotated each functional class by collecting all the sub-terms. We extracted all the functionally annotated proteins from the DisProt9.3 database. To obtain high-quality data, we removed sequences whose functional regions lacked annotations for disordered structure. We also excluded the DP00072 sequence that was too long (> 30,000 residues) to be processed by the protein language model. Subsequently, a total of 925 sequences were obtained and used for splitting the training, validation, and independent test datasets. Following the same protocols as previous studies [26, 32], we clustered the 925 sequences using the CD-HIT algorithm [60] with 25% sequence similarity. Then, we randomly divided the clusters into five subsets, where three subsets (including 589 sequences) were used as the training dataset, and one subset (including 148 sequences) was used as the validation dataset. The remaining subset with 188 sequences was used as the independent test dataset, namely DP93. To further evaluate the performance of the proposed predictor, we collected an additional independent test dataset (DP94) containing 98 sequences using the same protocol as aforementioned. The sequences of DP94 are collected from the newly updated proteins in versions 9.3 to 9.4 of the DisProt database. The statistical information of these datasets is shown in Additional file 1: Table S2.

### Graph-based interaction protein language model

Motivated by the language models (LMs) in natural language processing (NLP) [61, 62], the protein language models (PLMs) pre-trained with large numbers of amino acid sequences are able to discover the basic principles contained in the sequences [63]. Studies [55, 64, 65] have demonstrated that applying protein semantic information extracted from PLMs can facilitate the performance improvement of various prediction tasks. In DisoFLAG, we employed a graph-based interaction protein language model (GiPLM) to provide six functional predictions for intrinsically disordered regions (see Fig. 1b). The GiPLM integrates protein semantic information extracted from the ProtT5 [64] protein language model into graph-based interaction units to enhance the semantic correlation of multiple disordered functions. Specifically, a bidirectional gated recurrent neural network (Bi-GRU) [66] layer is employed to capture the protein contextual semantic encodings $\mathbf{P}$ based on the embeddings extracted from the ProtT5:

1 $$\mathbf{P}={\text{BiGRU}}({\mathbf{r}}_{1},{\mathbf{r}}_{2},\cdots ,{\mathbf{r}}_{L}]$$

where ${\mathbf{r}}_{i}$ is the PLM embedding vector for the *i*th residue, and *L* represents the length of the input sequence. Subsequently, the gate recurrent unit (GRU) layer with an attention mechanism [19, 67] was utilized to capture the global correlations among sequences and output the hidden representation ${\mathbf{h}}_{i}$ for each residue:

2 $${\mathbf{h}}_{i}={\text{GRU}}({\mathbf{h}}_{i-1},{\mathbf{c}}_{i})$$

3 $${\mathbf{c}}_{i}={\sum }_{j=1}^{l}{\alpha }_{ij}{\mathbf{p}}_{{\text{j}}}$$

4 $${\alpha }_{ij}=\frac{{\text{exp}}({s}_{ij})}{{\sum }_{j=1}^{l}{{\text{exp}}(s}_{ij})}$$

5 $${s}_{ij}={\mathbf{h}}_{i-1}^{T}{\mathbf{W}}_{a}{\mathbf{p}}_{{\text{j}}}$$

where ${\mathbf{p}}_{{\text{j}}}\in \mathbf{P}$ indicates the semantic encodings of the *j*th residue, ${\mathbf{W}}_{a}$ is the trainable weights of the attention mechanism, ${s}_{ij}$ the attention score between the *i*th and the *j*th residues, ${\alpha }_{ij}$ represents the attention weight between the *i*th and the *j*th residues, and ${\mathbf{c}}_{i}$ indicates the attention-based contextual representations. Then, the feature mapping layers were used to generate the functional semantic representations (**X**) for each residue. Specifically, six fully connected layers were employed for mapping the hidden global correlation representation ${\mathbf{h}}_{i}$ as functional semantic representations:

6 $${\mathbf{X}}_{i}^{(n)}={\text{ReLU}}\left({\mathbf{h}}_{i}{\mathbf{W}}^{\left(n\right)}+{\mathbf{b}}^{\left(n\right)}\right)$$

where ${\mathbf{X}}_{i}^{(n)}$ is the *nth* functional semantic representation for the *i*th residue and ${\mathbf{W}}^{\left(n\right)}$ and ${\mathbf{b}}^{(n)}$ are weights and bias variables, respectively; ReLU is the nonlinear activation function.

A single disordered region can bind to different ligands to perform multiple functions, and the multiple functions of IDRs are dependent and interrelated. In this study, we used the Shannon information entropy (IE) [68] and information gain (IG) [69] to describe the correlations among different functions:

7 $${IG}_{XY}={H}_{X\cup Y}- {H}_{XY }(0\le IG<1)$$

where ${H}_{X\cup Y}$ and ${H}_{XY}$ represent the information required for individual prediction and joint prediction of *X* and *Y* functions, respectively [68]:

8 $${H}_{X\cup Y}=-{\sum }_{i\in X\cup Y}p\left(i\right){{\text{log}}}_{2}p(i)$$

9 $${H}_{XY}=-{\sum }_{i\in X}{\sum }_{j\in Y}p\left(ij\right){{\text{log}}}_{2}p\left(ij\right)$$

A higher IG value indicates more reductions of IE in the joint prediction of two functions and a stronger correlation between the two functions. We pre-calculated the IG values on the training dataset and obtained the IG matrix of six disordered functions, which is visualized in Additional file 1: Fig. S1.

Then, each graph-based interaction unit in GiPLM models six disordered functions and their correlations as a functional graph G = (**V**, **E**), where **V** and **E** represent nodes and edges, respectively. The functional graph is fully connected (see Fig. 1b). Each node represents a function and is represented by functional semantic representation ${\mathbf{X}}^{\left(i\right)}$. Edges represent the correlations between functions and are represented by the adjacency matrix. In GiPLM, we employed a trainable weighted adjacency matrix to represent the degree of correlation between different functions and used the IG matrix pre-calculated on the training dataset by formula (7) as the initialization value:

10 $${A}_{ij}={IG}_{ij}$$

Then, the graph convolutional network (GCN) layer was used to propagate and aggregate the semantic correlation features for each node on the functional graph [70]:

11 $${\mathbf{Y}}_{i}^{(n)}={\text{ReLU}}({\sum }_{j\in {N}_{i}}{\mathbf{A}}_{ij}{\mathbf{W}}^{\left(n\right){\prime}}{\mathbf{X}}_{i}+{\mathbf{b}}^{\left(n\right){\prime}})$$

where ${\mathbf{Y}}_{i}^{(n)}$ is the aggregated semantic feature of the *n*th functional node, $\mathbf{A}$ is the trainable weighted adjacency matrix of the edges, ${\mathbf{X}}_{i}=[{\mathbf{X}}_{i}^{1},{\mathbf{X}}_{i}^{2},\cdots ,{\mathbf{X}}_{i}^{6}]$ is the concatenation of the six functional semantic representations, ${\mathbf{W}}^{\left(n\right){\prime}}$ is the convolution kernel, and ReLU is the nonlinear activation function. The semantic feature of the disorder ${\mathbf{Y}}_{i}^{IDR}$ was obtained by performing global max pooling over the functional graph (*F* represents the dimension of node features) [71]:

12 $${\mathbf{Y}}_{i}^{{\text{IDR}}}={{\text{max}}}_{k\in F}([{\mathbf{Y}}_{i}^{\left(1\right)},\cdots {\mathbf{Y}}_{i}^{\left(n\right)}\cdots ,{\mathbf{Y}}_{i}^{\left(6\right)}])$$

Finally, the propensity scores for disorder and six disordered functions were calculated based on the functional node features ${\mathbf{Y}}_{i}^{\left(1\right)\sim (6)}$ and disordered features ${\mathbf{Y}}_{i}^{{\text{IDR}}}$ by seven fully connected layers with the Sigmoid activation functions [32, 59].

### Model training and evaluation

To train the GiPLM model of DisoFLAG to predict disorder and disordered functions for proteins, we employed the binary cross-entropy loss function to calculate the loss value of each prediction, and their combination was used as the final loss *L*($\theta$) [72]:

13 $$L(\theta )=-\frac{1}{n+1}{\sum }_{i=1}^{n+1}[{y}_{i}{\text{log}}\left(\widehat{{y}_{i}}\right)+(1-{y}_{i}){\text{log}}(1-\widehat{{y}_{i}})]$$

where ${y}_{i}$ (1 or 0) and $\widehat{{y}_{i}}$ represent the trues and predicted propensity score of the *i*th function, respectively. All the model variables and hyper-parameters were optimized according to the minimum loss function values on the validation dataset. A detailed description of all the trainable parameters and hyper-parameters of DisoFLAG was given in Additional file 1: Table S3.

The DisoFLAG outputs the real-valued propensity score results for disorder and disordered functions. We evaluated the predictive performances of DisoFLAG and other comparative methods with threshold-independent metrics [73–77]: AUC (area under the true-positive rates and false-positive rates curve across all thresholds), AUPR (area under the precisions and recalls curve across all thresholds), APS (average precision score along the precision-recall curve), and ${F}_{{\text{max}}}$ (the maximum harmonic mean between precision and recall rate across all thresholds). In addition, given a threshold, the binary results can be converted from the real-valued results (residue is predicted to be disordered/functional if its propensity score is higher than the threshold; otherwise, it is predicted as ordered/non-functional). We used the Matthews correlation coefficient (MCC) and balanced accuracy (BACC) to evaluate the binary prediction results. The definitions of the evaluation metrics are given in Additional file 1: Table S4.

## Results and discussion

### Protein semantic information facilitates the prediction of intrinsic disorder and disordered function

**Fig. 2:**
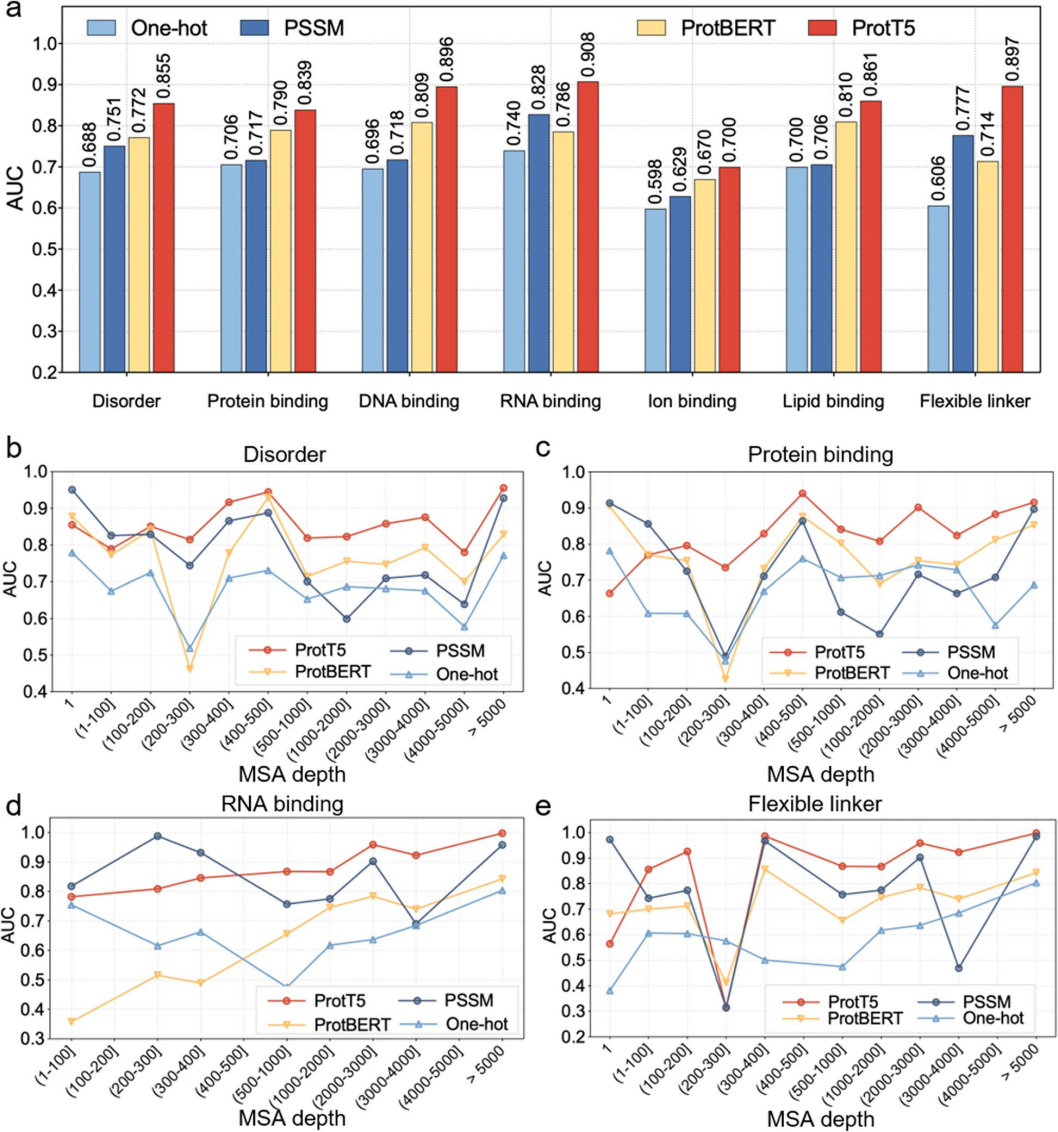
Performance of DisoFLAG in predicting disorder and disordered functions using different feature representations. **a** AUC value comparisons of DisoFLAG using different features, including protein language model-based features (ProtT5 and ProtBERT) and classic protein feature representations by position-specific scoring matrix (PSSM) and amino acid one-hot encodings (One-hot). The performance of DisoFLAG in predicting disorder (**b**) and disordered functions (**c**–**e**) for sequences with different multiple sequence alignment (MSA) depths

Protein feature representation is an essential step in DisoFLAG. We evaluated the performance of DisoFLAG using different protein representations, including protein language model-based (PLM) features (ProtT5 and ProtBERT), the position-specific scoring matrix (PSSM), and amino acid one-hot encodings (One-hot). Models taking different feature inputs were trained and optimized following the same protocol as described in the “Methods” section. The evaluation results on the DP93 independent test dataset and corresponding ranking results are shown in Fig. 2a and Additional file 1: Table S5, respectively. From these results, we can see that the model using PLM-based features outperformed PSSM and One-hot, and the model using ProtT5 consistently achieved the highest performance in predicting disorder and disordered functions. To further investigate the model performance improvement by the PLM-based features, we calculated the AUC values of DisoFLAG on the sequences with different multiple sequence alignment (MSA) [78, 79] depths (see Fig. 2b–e). Specifically, for each sequence in the DP93 dataset, we employed the HHblits [80] tools to conduct homology searches against the UniProt database and grouped the sequences according to the number of rows in the MSA search results. The results on disorder (Fig. 2b) and disordered functions (Fig. 2c–e) demonstrated that the performance of the model using the protein language model encodings improved the most as the number of sequence homologous alignments (i.e., MSA depth) increased. When the MSA depth is relatively small, the PSSM encoding method has better results than the protein language model coding method. The possible reasons for these results were attributed to the following: (1) The PLM as a data-driven deep learning method can accurately capture sequence features only when there is a sufficient number of homologous sequences available. (2) In contrast, PSSM encoding based on a probabilistic statistical model is more effective in capturing sequence features under a lower MSA depth condition. (3) The features captured by PSSM encoding and PLM are different. PSSM is designed to encode sequence conservation information, while PLM learns the contextual semantic information of protein sequences. Therefore, the conservation information is more accurate than the semantic information in predicting disordered functions when there are fewer homologous sequences.

### Graph-based interaction unit enhances the semantic correlations of multiple disordered functions

**Fig. 3:**
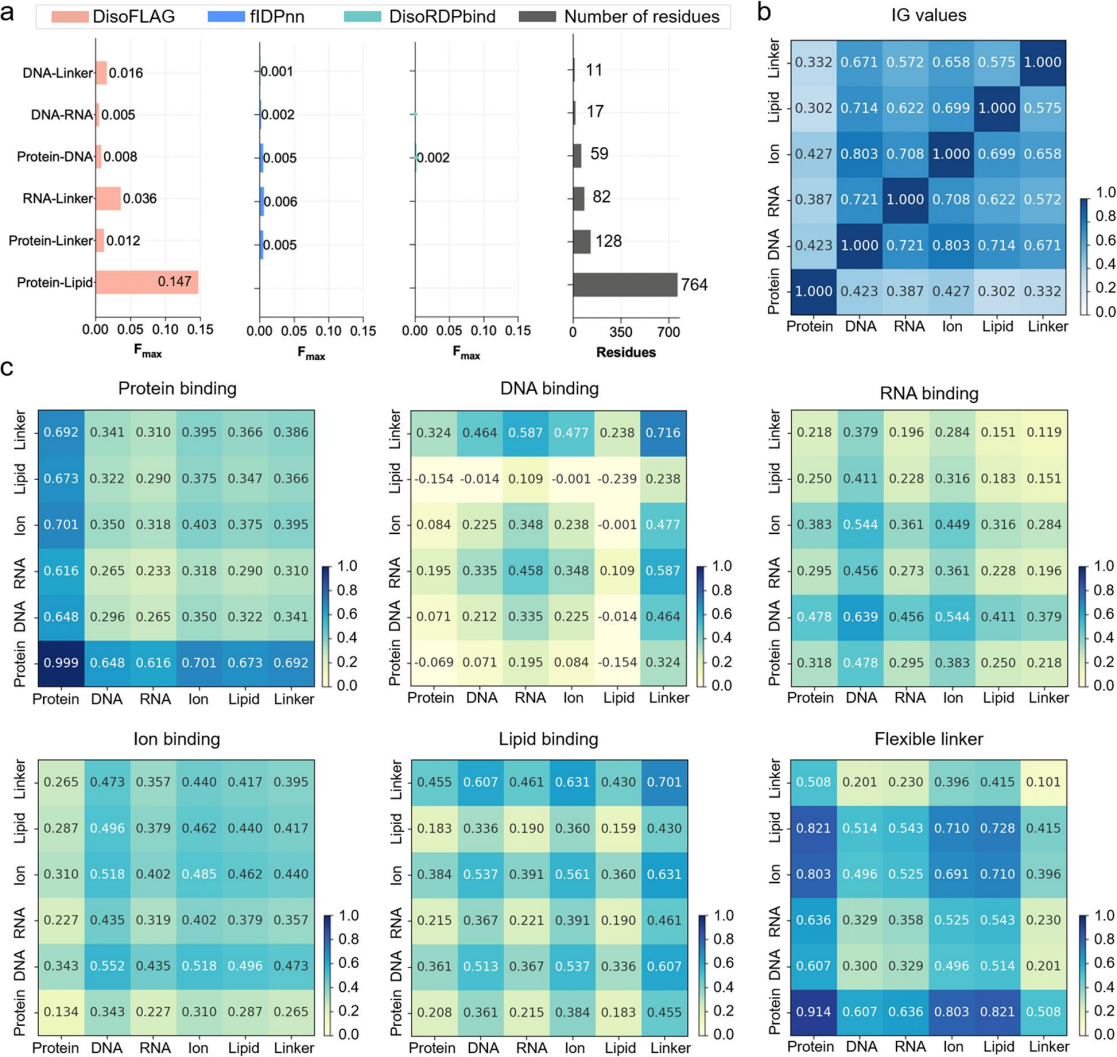
Functional correlations contribute to the prediction of disordered functions. **a** Performance comparison of different predictors on multifunctional residues, “/” represents the predictor failed to process this subset of residues. The information gain (IG) values calculated on the DP93 test dataset (**b**), and their contributions (**c**) to the prediction of different functions calculated by layer-wise relevance propagation (LRP)

The graph-based interaction unit (GiU) in DisoFLAG was employed to establish the correlations among multiple disordered functions. To investigate the critical role of GiU in DisoFLAG, we compared the performance of DisoFLAG using GiU with a simple sequence layer (Seq) (see Table 1). From this table, we found that DisoFLAG using GiU consistently outperformed the Seq, indicating that the semantic correlation features captured by GiU significantly boosted the predictive performance of DisoFLAG. In addition, the correlation among different disordered functions leads to one disordered residue being able to perform two or more different functions, which is referred to as the multifunctional (MF) residue. We compared DisoFLAG with other methods for predicting MF residues on the DP93 test dataset. A multifunctional residue is considered correctly predicted only if all its functions are accurately predicted. The *F*_max_ evaluation results of different methods are shown in Fig. 3a, from which we found that there are six types of MF residues in the DP93 dataset. DisoFLAG is the only predictor that can predict all types of MF residues. Additionally, compared to other predictors, DisoFLAG considered correlations among different functions and achieved the highest *F*_max_ values, which again indicated the importance of functional correlations captured by GiU for the accurate prediction of disordered functions.

**Table 1:** Performance comparisons of DisoFLAG using graph-based interaction unit (GiU) and sequence layer (Seq) for predicting different disordered functions on the DP93 independent test dataset

	**Module**	**AUC**	**AUPR**	***F***_**max**_	**MCC**	**BACC**
Protein binding	GiU	0.839	0.768	0.434	0.370	0.768
	Seq^a^	0.827	0.752	0.411	0.339	0.752
DNA binding	GiU	0.896	0.821	0.152	0.181	0.821
	Seq^a^	0.836	0.775	0.077	0.111	0.775
RNA binding	GiU	0.908	0.850	0.207	0.222	0.850
	Seq^a^	0.838	0.764	0.160	0.164	0.764
Ion binding	GiU	0.700	0.695	0.028	0.069	0.695
	Seq^a^	0.637	0.654	0.029	0.058	0.654
Lipid binding	GiU	0.861	0.771	0.304	0.328	0.771
	Seq^a^	0.830	0.757	0.135	0.161	0.757
Flexible linker	GiU	0.897	0.833	0.403	0.389	0.833
	Seq^a^	0.769	0.709	0.118	0.134	0.709

^a^DisoFLAG using the sequence decoder unit (Seq) is achieved by removing the graph-based interaction units and GCN layer from the GiPLM architecture

Furthermore, we used layer-wise relevance propagation (LRP) [81, 82] to investigate the contributions of functional correlations to the prediction results. The LRP score was calculated as follows:

14 $${{\text{R}}}_{j}^{(l)}=(\alpha \frac{{w}_{jk}^{+}{h}_{j}}{{\sum }_{j}{w}_{jk}^{+}{h}_{j}+{b}_{k}^{+}}-\beta \frac{{w}_{jk}^{-}{h}_{j}}{{\sum }_{j}{w}_{jk}^{-}{h}_{j}+{b}_{k}^{-}}){{\text{R}}}_{k}^{(l+1)}$$

where ${{\text{R}}}_{j}^{(l)}$ and ${{\text{R}}}_{k}^{(l+1)}$ are the relevance scores of the current and previous layers, respectively. $\alpha$ and $\beta$ are the constraint parameters of the $\alpha \beta$ rule in LRP; ${w}_{jk}$, ${b}_{k}$, and ${h}_{j}$ represent the weights, bias, and hidden vector, respectively. We performed the LRP on the graph-based interaction units to obtain the importance of functional correlations. For each function, the importance scores of functional correlations to the propensity score were calculated by summing the relevance scores of all true-positive propensity predictions on the DP93 test dataset, which were described in Eq. (14). Figure 3b shows the IG values calculated on the DP93 dataset, which reflected the correlation among different functions. These correlations consistently made a positive contribution to the prediction of six disordered functions (see Fig. 3c).

### Comparison of DisoFLAG to other methods in the prediction of disordered functions

We evaluated the performance of DisoFLAG for predicting disordered functions and compared it with methods specifically designed for disordered functions and performed well on CAID2. These methods include DisoRDPbind [29] and DeepDISOBind [30] for predicting protein-, DNA-, and RNA-binding disordered regions (IDRs); fIDPnn [26] method for predicting protein-, DNA-, and RNA-binding IDRs and disordered linkers; ANCHOR-2 [27] for predicting protein-binding IDRs; MoRFchibi (Light and Web) [34] and SPOT-MoRF [33] are methods for identifying molecular recognition features (MoRFs), which are protein-binding IDRs that undergo a disorder-to-order conformational transition; DisoLipPred [32] is the only available method for predicting lipid-binding IDRs; and TransDFL [39] and DFLpred [38] are methods for identification of disordered linkers. DisoFLAG is currently the only predictor providing predictions of ion-binding IDRs and covering the broadest range of disordered functional categories. The evaluation results on the DP93 test dataset suggested that the performance of DisoFLAG in predicting disordered protein-binding, DNA-binding, RNA-binding, lipid-binding, and linkers is better than the current tools quantified by AUC, MCC, and BACC metrics (Table 2). Moreover, DisoFLAG offered statistically significant improvement in AUC compared to other methods (see Additional file 1: Table S6). To further investigate the stability of the prediction performance of different methods, we performed the performance comparison on the DP94 test dataset, whose proteins were collected from the latest version 9.4 DisProt database. The results show that the performance quantified by the AUC metric of DisoFLAG is still significantly better than current tools in predicting disordered protein-binding, DNA-binding, and linkers; however, its performance decreased in predicting RNA-binding and lipid-bindings (see Additional file 1: Tables S7 and S8). We also reported the performance metrics at the protein level, as described in Additional file 1: Tables S9 and S10.

**Table 2:** Performance comparisons of DisoFLAG and other predictors on the DP93 independent test dataset

**Prediction**	**Method**^a^	**AUC**	**AUPR**	***F***_**max**_	**MCC**	**BACC**
Protein binding	DisoFLAG	0.839	0.340	0.434	0.370	0.768
	fIDPnn^b^	0.817	0.277	0.427	0.357	0.758
	DeepDISOBind^b^	0.808	0.384	0.438	0.361	0.727
	DisoRDPbind^b^	0.780	0.243	0.395	0.335	0.752
	ANCHOR-2^c^	0.741	0.222	0.359	0.277	0.705
	MoRFchibi-Light^b^	0.729	0.269	0.311	0.210	0.664
	SPOT-MoRF^c^	0.721	0.223	0.296	0.207	0.658
	MoRFchibi-Web^b^	0.688	0.243	0.280	0.173	0.629
DNA binding	DisoFLAG	0.896	0.053	0.152	0.181	0.821
	fIDPnn^b^	0.812	0.069	0.160	0.161	0.805
	DisoRDPbind^b^	0.703	0.035	0.125	0.124	0.663
	DeepDISOBind^b^	0.696	0.010	0.025	0.060	0.689
RNA binding	DisoFLAG	0.908	0.127	0.207	0.222	0.850
	DeepDISOBind^b^	0.823	0.163	0.338	0.327	0.765
	fIDPnn^b^	0.816	0.061	0.126	0.190	0.797
	DisoRDPbind^b^	0.526	0.019	0.044	0.027	0.541
Ion binding	DisoFLAG	0.700	0.013	0.028	0.069	0.695
Lipid binding	DisoFLAG	0.861	0.251	0.304	0.328	0.771
	DisoLipPred^b^	0.644	0.029	0.070	0.065	0.615
Flexible linker	DisoFLAG	0.897	0.273	0.403	0.389	0.833
	TransDFL^c^	0.781	0.221	0.206	0.166	0.730
	fIDPnn^b^	0.712	0.046	0.093	0.102	0.666
	DFLpred^b^	0.635	0.046	0.095	0.081	0.615

^a^The evaluation results of the comparative methods were calculated based on the results obtained by running their respective web servers^b^ and standalone packages^c^. Predictors in each prediction are sorted by their AUC value

**Fig. 4:**
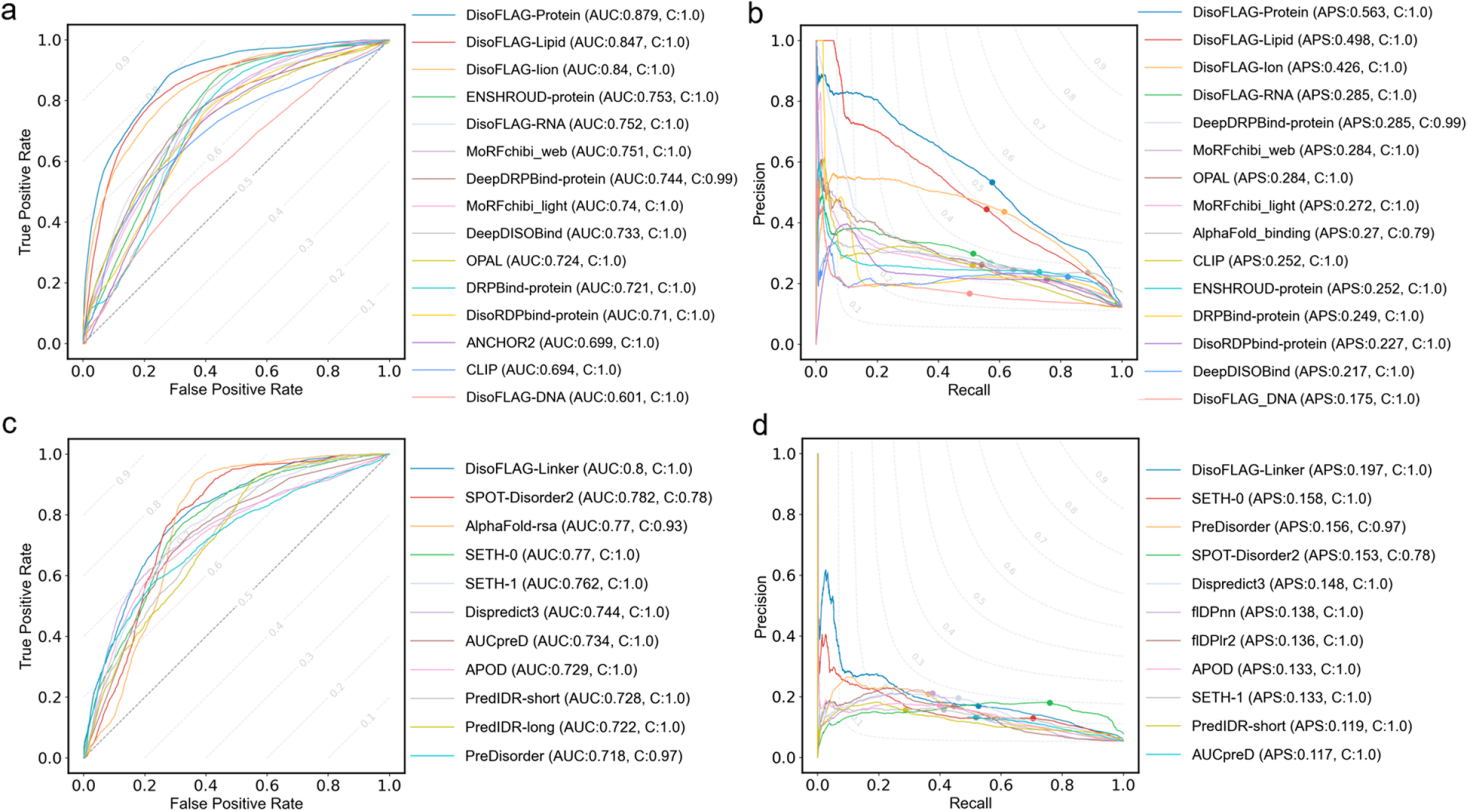
Performance comparisons of DisoFLAG and the 10 top-ranking methods in CAID2 for disordered binding and linker prediction. The receiver operating characteristic (ROC) curves on the disorder-binding and disorder-linker predictions are shown in **a** and **c**, respectively; methods are sorted by the area under the ROC cover (AUC). The precision-recall (PR) curves on the disorder-binding and disorder-linker predictions are shown in **b** and **d**, respectively; methods are sorted by the average precision score (APS); and points correspond to the *F*_max_ values. “C” represents the coverage of prediction results

We compared DisoFLAG with a broad range of predictors that participated in the Critical Assessment of protein Intrinsic Disorder (CAID2) challenge. Specifically, we assessed the performance of DisoFLAG on two CAID2 test datasets: disorder-binding and disorder-linker [22, 23]. The disorder-binding dataset contains 78 proteins annotated with interaction interfaces in disordered regions, and the disorder-linker dataset contains 40 proteins with disordered flexible linkers. We comprehensively aligned the sequences in CAID2 with all the benchmark datasets used in this study and found that CAID2 sequences were completely unseen with the training and validation of DisoFLAG. This is fully consistent with the assessment process of CAID2. Therefore, it is appropriate to directly compare the results of DisoFLAG with those reported in CAID2. We assessed the performance of DisoFLAG for predicting protein-binding, DNA-binding, RNA-binding, ion-binding, and lipid-binding on the disorder-binding dataset and predicting linkers on the disorder-linker dataset. The evaluation results and comparison with the 10 top-ranking methods reported in CAID2 [22, 23] are shown in Fig. 4. In Fig. 4a, b, we can see that the DisoFLAG’s protein-binding predictor generates the highest quality predictions with AUC = 0.879 and APS = 0.563 on the disorder-binding dataset. The DisoFLAG’s linker predictor achieves AUC = 0.8 and APS = 0.197 for the prediction of disordered linkers on the disorder-linker dataset (see Fig. 4c, d). The complete metrics are listed in Additional file 1: Tables S11 and S12.

### Comparison of DisoFLAG to other methods in the prediction of intrinsic disorder

**Fig. 5:**
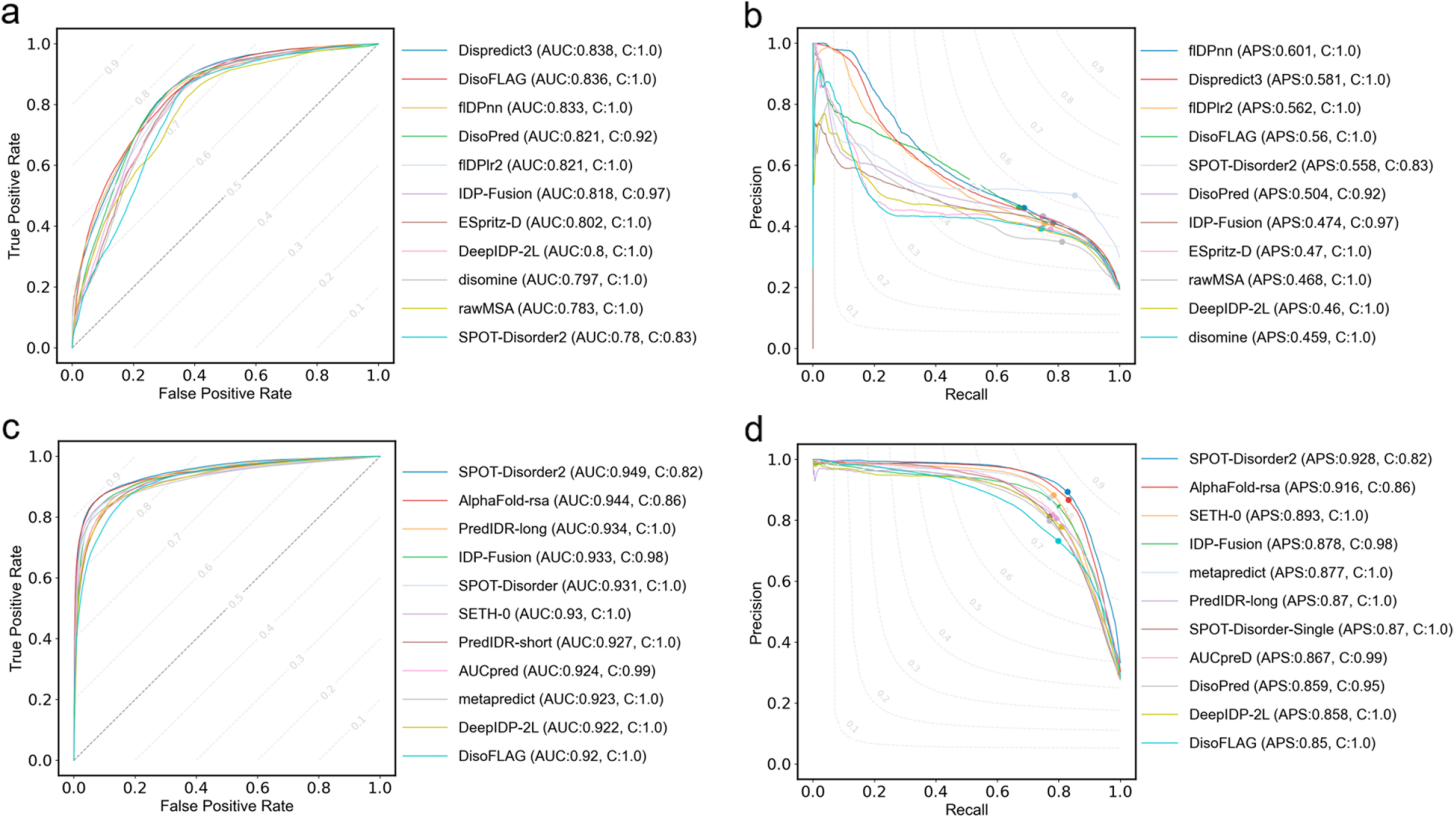
Performance comparisons of DisoFLAG and the 10 top-ranking methods in CAID2 for disorder prediction. The receiver operating characteristic (ROC) curves on the Disorder-NOX (210 proteins) and Disorder-PDB (348 proteins) datasets are shown in **a** and **c**, respectively, and methods are sorted by the area under ROC cover (AUC). The precision-recall (PR) curves on the Disorder-NOX and Disorder-PDB datasets are shown in **b** and **d**, respectively; methods are sorted by the average precision score (APS); and points correspond to the *F*_max_ values. “C” represents the coverage of prediction results

We assessed the performance of DisoFLAG in predicting the intrinsic disorder of proteins on two disordered test datasets provided in CAID2: DisProt-NOX and DisProt-PDB. The DisProt-NOX dataset is composed of IDRs from the DisProt database, excluding X-ray missing residues. In contrast, the DisProt-PDB dataset is more conservative by strictly limiting negative samples to structured residues observed in the PDB database. For more detailed information about the datasets, please refer to the CAID2 [22, 23]. We performed a thorough sequence comparison of two CAID2 datasets against the benchmark dataset used in this study to ensure that all sequences were independent and unseen by the training and validation of DisoFLAG. Subsequently, we compared the performance of DisoFLAG with the top 10 ranked methods reported in CAID2 (see Fig. 5). From these results, we observed that DisoFLAG achieved a second rank with an AUC of 0.836 and a fourth rank with an APS of 0.560 on the Disorder-NOX dataset. DisoFLAG showed lower performance on the Disorder-PDB dataset, but it achieved comparable performance to the CAID2 top 10 results in terms of AUC and APS metrics. The complete metrics are in Additional file 1: Tables S13 and S14.

### Case study

**Fig. 6:**
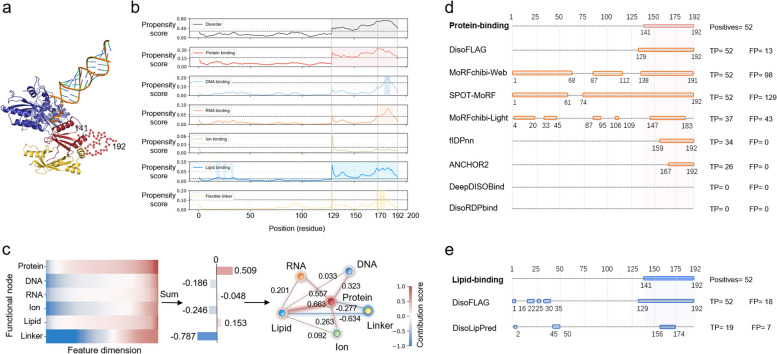
Prediction results of DisoFLAG for Vif protein. **a** Protein complex structure (PDB ID: 8E40) of Vif (colored in red), A3G (colored in blue), CBF-beta (colored in yellow), and fork RNA (colored in orange). **b** The propensity score results predicted by DisoFLAG for the Vif protein. **c** LRP of residue T170’s protein-binding propensity score on the functional graph, where the contribution scores of nodes were calculated by summing the relevance scores of node features, and the contribution score of the edge was equal to the sum of contributions of two nodes it links. The binary results of protein binding (**d**) and lipid binding (**e**) predicted by DisoFLAG and other methods for the Vif protein. The binary results were converted from the propensity scores of different methods using a threshold that achieves the maximum F1 score

We investigated the prediction results of DisoFLAG for one protein from the independent test data: the human immunodeficiency virus infectivity factor (HIV-1 Vif, DisProt: DP00875). Vif is a crucial accessory protein in HIV replication, and its role is to disrupt the antiviral activity of the human host defense factor APOBEC-3G (A3G) [83]. The functional implementation of Vif involves interactions with A3G, protein chaperones, ubiquitination machinery factors, and so on [84, 85]. Thus, elucidating the functional mechanism of Vif is of significant importance for discovering novel drugs to block its activity [85–87]. Nuclear magnetic resonance (NMR) revealed that the C-terminal domain (141–192) of Vif is unstructured under physiological conditions. Figure 6a shows a protein complex structure (PDB ID: 8E40) [88] composed of Vif from the PDB database [89]. Experimental evidence suggests that the disordered region of Vif is involved in binding with proteins and lipids [90]. The propensity scores for the Vif protein produced by DisoFLAG are visualized in Fig. 6b. To investigate the contribution of functional correlations to the DisoFLAG’s predictions, we mapped the highest protein-binding propensity score located at the T170 residue onto the functional graph in DisoFLAG. The mapping process achieved by LRP is shown in Fig. 6c, from which we observed that protein-binding, RNA-binding, and lipid-binding nodes made a positive contribution to the prediction, and the edge between the protein-binding node and lipid-binding node contributed the most. We further compared the binary results of protein-binding and lipid-binding predicted by DisoFLAG and other methods for the Vif protein. From the comparison results shown in Fig. 6d, e, it can be seen that DisoFLAG is the only method that can simultaneously identify the complete disordered protein-binding and lipid-binding regions of the Vif protein and has the lowest number of false-positive predictions. These results highlighted again the semantic correlations captured through the graph-based interaction protein language model (GiPLM) enabling DisoFLAG to provide accurate and comprehensive predictions of multiple disordered functions.

## Conclusions

Inspired by the similarities between biological sequences and natural language across three hierarchical levels, we designed the DisoFLAG predictor based on a graph-based interaction protein language model. DisoFLAG provides predictions of intrinsic disorder and its six types of functions, including protein-binding, DNA-binding, RNA-binding, ion-binding, lipid-binding, and flexible linkers. The performance assessments performed on two independent test datasets and CIAD2 benchmark test datasets indicated that DisoFLAG offers accurate and comprehensive predictions of disordered functions, extending the current coverage of computationally predicted disordered function categories. Our experimental analysis of the prediction results of DisoFLAG demonstrated that the use of protein semantic knowledge extracted from the pre-trained protein language model facilitated the accurate predictions of multiple disordered functions. The graph-based interaction unit used in DisoFLAG enhanced the semantic relevance of multiple disordered functions leading to a significant improvement in the identification of multifunctional disordered residues. We provide the standalone package and a convenient web server for DisoFLAG, which will be helpful tools to researchers in related fields.

### Supplementary Information

**Additional file 1: Fig. S1.** Visualization of the IG matrix. **Table S1.** The ontology term and its sub-terms for each disordered functional class. **Table S2.** The statistical information of the datasets. **Table S3.** The number of trainable variables and hyper-parameters of DisoFLAG. **Table S4.** The definition of evaluation metrics. **Table S5.** The performance ranking of DisoFLAG using different features. **Table S6.** The statistical significance of differences (*p*-value) in predictive performance by different methods on the DP93 test dataset. **Table S7.** Performance comparisons of DisoFLAG and other predictors on the DP94 independent test dataset. **Table S8.** The statistical significance of differences (*p*-value) in predictive performance by different methods on the DP94 test dataset. **Table S9.** Per-protein performance of different disordered function predictors on the DP93 test dataset. **Table S10.** Per-protein performance of different disordered function predictors on the DP94 test dataset. **Table S11.** Performance metrics for Disorder-Binding prediction on the CAID2 test dataset. **Table S12.** Performance metrics for Disorder-Linker prediction on the CAID2 test dataset. **Table S13.** Performance metrics for disorder prediction on the CAID2 Disorder-NOX and Disorder-PDB test datasets. **Table S14.** Per-protein performance of different disorder predictors on the CAID2 Disorder-NOX and Disorder-PDB test datasets.

**Additional file 2. **The data values for the figures.

## Data Availability

All data generated or analyzed during this study are included in this published article, its supplementary information files, and publicly available repositories. All the benchmark datasets used in this study and prediction results of the methods involved in the evaluation conducted of this study are available in the Zenodo repository (https://doi.org/10.5281/zenodo.10361856). The source code and its descriptions of DisoFLAG are reproducible in the GitHub repository (https://github.com/YihePang/DisoFLAG), which is archived on Zenodo at https://doi.org/10.5281/zenodo.10360345. The data values for the figures are provided in the Additional file 2. The web server of DisoFLAG can be accessed from http://bliulab.net/DisoFLAG/.

## Abbreviations

IDP/IDR: Intrinsically disordered protein and region
GiPLM: Graph-based interaction protein language model
CAID: Critical Assessment of protein Intrinsic Disorder
MoRF: Molecular recognition feature
DFL: Disordered flexible linker
NLP: Natural language processing
LM: Language model
PLM: Protein language model
GCN: Graph convolutional network
IG: Information gain
GRU: Gated recurrent unit
Bi-GRU: Bi-directional gated recurrent unit
IDPO: Intrinsically Disordered Proteins Ontology
GO: Gene Ontology
ROC: Receiver operating characteristic curve
AUC: Area under the ROC curve
PR: Precision-recall curve
AUPR: Area under the PR curve
APS: Average precision score
MCC: Matthews correlation coefficient
BACC: Balanced accuracy
PSSM: Position-specific scoring matrix
MSA: Multiple sequence alignment
GiU: Graph-based interaction unit
LRP: Layer-wise relevance propagation
HIV-1 Vif: Human immunodeficiency virus infectivity factor
A3G: APOBEC-3G protein
CBF-beta: Core-binding factor subunit beta protein
NMR: Nuclear magnetic resonance

## References

[1] Iakoucheva LM, Brown CJ, Lawson JD, Obradovic Z, Dunker AK (2002). Intrinsic disorder in cell-signaling and cancer-associated proteins. J Mol Biol.

[2] Wright PE, Dyson HJ (2015). Intrinsically disordered proteins in cellular signalling and regulation. Nat Rev Mol Cell Biol.

[3] Zhou J, Zhao S, Dunker AK (2018). Intrinsically disordered proteins link alternative splicing and post-translational modifications to complex cell signaling and regulation. J Mol Biol.

[4] Uversky VN, Oldfield CJ, Dunker AK (2008). Intrinsically disordered proteins in human diseases: introducing the D2 concept. Annu Rev Biophys.

[5] Eftekharzadeh B, Daigle JG, Kapinos LE, Coyne A, Schiantarelli J, Carlomagno Y, Cook C, Miller SJ, Dujardin S, Amaral AS (2018). Tau protein disrupts nucleocytoplasmic transport in Alzheimer’s disease. Neuron.

[6] Haass C, Selkoe DJ (2007). Soluble protein oligomers in neurodegeneration: lessons from the Alzheimer’s amyloid beta-peptide. Nat Rev Mol Cell Biol.

[7] Jaikaran ET, Higham CE, Serpell LC, Zurdo J, Gross M, Clark A, Fraser PE (2001). Identification of a novel human islet amyloid polypeptide beta-sheet domain and factors influencing fibrillogenesis. J Mol Biol.

[8] Tang W, Wan S, Yang Z, Teschendorff AE, Zou Q (2018). Tumor origin detection with tissue-specific miRNA and DNA methylation markers. Bioinformatics.

[9] Cheng Y, LeGall T, Oldfield CJ, Dunker AK, Uversky VN (2006). Abundance of intrinsic disorder in protein associated with cardiovascular disease. Biochemistry.

[10] Cao C, Wang J, Kwok D, Cui F, Zhang Z, Zhao D, Li MJ, Zou Q (2022). webTWAS: a resource for disease candidate susceptibility genes identified by transcriptome-wide association study. Nucleic Acids Res.

[11] Zeng X, Xiang H, Yu L, Wang J, Li K, Nussinov R (2022). Cheng FJNMI: Accurate prediction of molecular properties and drug targets using a self-supervised image representation learning framework. Nat Mach Intell.

[12] Cheng Y, LeGall T, Oldfield CJ, Mueller JP, Van YY, Romero P, Cortese MS, Uversky VN, Dunker AK (2006). Rational drug design via intrinsically disordered protein. Trends Biotechnol.

[13] Zeng X, Wang F, Luo Y (2022). Kang S-g, Tang J, Lightstone FC, Fang EF, Cornell W, Nussinov R, Cheng FJCRM: Deep generative molecular design reshapes drug discovery. Cell Rep Med.

[14] UniProt C (2023). UniProt: the universal protein knowledgebase in 2023. Nucleic Acids Res.

[15] Hanson J, Yang Y, Paliwal K, Zhou Y (2017). Improving protein disorder prediction by deep bidirectional long short-term memory recurrent neural networks. Bioinformatics.

[16] Jones DT, Cozzetto D (2015). DISOPRED3: precise disordered region predictions with annotated protein-binding activity. Bioinformatics.

[17] Zhang T, Faraggi E, Xue B, Dunker AK, Uversky VN, Zhou Y (2012). SPINE-D: accurate prediction of short and long disordered regions by a single neural-network based method. J Biomol Struct Dyn.

[18] Wang S, Ma J, Xu J (2016). AUCpreD: proteome-level protein disorder prediction by AUC-maximized deep convolutional neural fields. Bioinformatics.

[19] Tang YJ, Pang YH, Liu B (2021). IDP-Seq2Seq: identification of intrinsically disordered regions based on sequence to sequence learning. Bioinformatics.

[20] Hanson J, Paliwal KK, Litfin T, Zhou Y (2019). SPOT-Disorder2: improved protein intrinsic disorder prediction by Ensembled deep learning. Genom Proteom Bioinf.

[21] Necci M, Piovesan D, Predictors C, DisProt C, Tosatto SCE (2021). Critical assessment of protein intrinsic disorder prediction. Nat Methods.

[22] Conte AD, Mehdiabadi M, Bouhraoua A, Miguel Monzon A, Tosatto SCE, Piovesan D (2023). Critical assessment of protein intrinsic disorder prediction (CAID) - results of round 2. Proteins.

[23] Del Conte A, Bouhraoua A, Mehdiabadi M, Clementel D, Monzon AM (2023). predictors C, Tosatto SCE, Piovesan D: CAID prediction portal: a comprehensive service for predicting intrinsic disorder and binding regions in proteins. Nucleic Acids Res.

[24] Tompa P (2002). Intrinsically unstructured proteins. Trends Biochem Sci.

[25] van der Lee R, Buljan M, Lang B, Weatheritt RJ, Daughdrill GW, Dunker AK, Fuxreiter M, Gough J, Gsponer J, Jones DT (2014). Classification of intrinsically disordered regions and proteins. Chem Rev.

[26] Hu G, Katuwawala A, Wang K, Wu Z, Ghadermarzi S, Gao J, Kurgan L (2021). flDPnn: accurate intrinsic disorder prediction with putative propensities of disorder functions. Nat Commun.

[27] Dosztanyi Z, Meszaros B, Simon I (2009). ANCHOR: web server for predicting protein binding regions in disordered proteins. Bioinformatics.

[28] Meszaros B, Erdos G, Dosztanyi Z (2018). IUPred2A: context-dependent prediction of protein disorder as a function of redox state and protein binding. Nucleic Acids Res.

[29] Peng Z, Kurgan L (2015). High-throughput prediction of RNA, DNA and protein binding regions mediated by intrinsic disorder. Nucleic Acids Res.

[30] Zhang F, Zhao B, Shi W, Li M, Kurgan L (2022). DeepDISOBind: accurate prediction of RNA-, DNA- and protein-binding intrinsically disordered residues with deep multi-task learning. Brief Bioinform.

[31] Meszaros B, Simon I, Dosztanyi Z (2009). Prediction of protein binding regions in disordered proteins. PLoS Comput Biol.

[32] Katuwawala A, Zhao B, Kurgan L (2021). DisoLipPred: accurate prediction of disordered lipid-binding residues in protein sequences with deep recurrent networks and transfer learning. Bioinformatics.

[33] Hanson J, Litfin T, Paliwal K, Zhou Y (2020). Identifying molecular recognition features in intrinsically disordered regions of proteins by transfer learning. Bioinformatics.

[34] Malhis N, Jacobson M, Gsponer J (2016). MoRFchibi SYSTEM: software tools for the identification of MoRFs in protein sequences. Nucleic Acids Res.

[35] Disfani FM, Hsu WL, Mizianty MJ, Oldfield CJ, Xue B, Dunker AK, Uversky VN, Kurgan L (2012). MoRFpred, a computational tool for sequence-based prediction and characterization of short disorder-to-order transitioning binding regions in proteins. Bioinformatics.

[36] Sorensen CS, Kjaergaard M (2019). Effective concentrations enforced by intrinsically disordered linkers are governed by polymer physics. Proc Natl Acad Sci U S A.

[37] Anand S, Mohanty D (2012). Inter-domain movements in polyketide synthases: a molecular dynamics study. Mol Biosyst.

[38] Meng F, Kurgan L (2016). DFLpred: high-throughput prediction of disordered flexible linker regions in protein sequences. Bioinformatics.

[39] Pang Y, Liu B (2023). TransDFL: identification of disordered flexible linkers in proteins by transfer learning. Genom Proteom Bioinf.

[40] Peng Z, Xing Q, Kurgan L (2020). APOD: accurate sequence-based predictor of disordered flexible linkers. Bioinformatics.

[41] Enard W, Przeworski M, Fisher SE, Lai CS, Wiebe V, Kitano T, Monaco AP, Paabo S (2002). Molecular evolution of FOXP2, a gene involved in speech and language. Nature.

[42] Darwin C: The descent of man, and selection in relation to sex, vol. 1: Murray; 1888.

[43] Searls DB (2002). The language of genes. Nature.

[44] Strait BJ, Dewey TG (1996). The Shannon information entropy of protein sequences. Biophys J.

[45] Wang R, Jiang Y, Jin J, Yin C, Yu H, Wang F, Feng J, Su R, Nakai K, Zou Q (2023). DeepBIO: an automated and interpretable deep-learning platform for high-throughput biological sequence prediction, functional annotation and visualization analysis. Nucleic Acids Res.

[46] Zhang W, Meng Q, Wang J, Guo F (2022). HDIContact: a novel predictor of residue-residue contacts on hetero-dimer interfaces via sequential information and transfer learning strategy. Brief Bioinform.

[47] Meng Q, Guo F, Wang E, Tang J (2023). ComDock: a novel approach for protein-protein docking with an efficient fusing strategy. Comput biol med.

[48] Rives A, Meier J, Sercu T, Goyal S, Lin Z, Liu J, Guo D, Ott M, Zitnick CL, Ma J (2021). Biological structure and function emerge from scaling unsupervised learning to 250 million protein sequences. Proc Natl Acad Sci U S A.

[49] Li H, Pang Y, Liu B (2021). BioSeq-BLM: a platform for analyzing DNA, RNA, and protein sequences based on biological language models. Nucleic Acids Res.

[50] Jin J, Yu Y, Wang R, Zeng X, Pang C, Jiang Y, Li Z, Dai Y, Su R, Zou Q (2022). iDNA-ABF: multi-scale deep biological language learning model for the interpretable prediction of DNA methylations. Genome biol.

[51] Bepler T, Berger B (2021). Learning the protein language: evolution, structure, and function. Cell Syst.

[52] Ferruz N, Schmidt S, Hocker B (2022). ProtGPT2 is a deep unsupervised language model for protein design. Nat Commun.

[53] Madani A, Krause B, Greene ER, Subramanian S, Mohr BP, Holton JM, Olmos JL, Xiong C, Sun ZZ, Socher R (2023). Large language models generate functional protein sequences across diverse families. Nat Biotechnol.

[54] Chen L, Yu L, Gao L (2023). Potent antibiotic design via guided search from antibacterial activity evaluations. Bioinformatics.

[55] Unsal S, Atas H, Albayrak M, Turhan K, Acar AC, Doğan T (2022). Learning functional properties of proteins with language models. Nat Mach Intell.

[56] Hatos A, Hajdu-Soltesz B, Monzon AM, Palopoli N, Alvarez L, Aykac-Fas B, Bassot C, Benitez GI, Bevilacqua M, Chasapi A (2020). DisProt: intrinsic protein disorder annotation in 2020. Nucleic Acids Res.

[57] Piovesan D, Tabaro F, Micetic I, Necci M, Quaglia F, Oldfield CJ, Aspromonte MC, Davey NE, Davidovic R, Dosztanyi Z (2017). DisProt 7.0: a major update of the database of disordered proteins. Nucleic Acids Res.

[58] Quaglia F, Meszaros B, Salladini E, Hatos A, Pancsa R, Chemes LB, Pajkos M, Lazar T, Pena-Diaz S, Santos J (2022). DisProt in 2022: improved quality and accessibility of protein intrinsic disorder annotation. Nucleic Acids Res.

[59] Pang Y, Liu B (2022). DMFpred: predicting protein disorder molecular functions based on protein cubic language model. PLoS Comput Biol.

[60] Huang Y, Niu B, Gao Y, Fu L, Li W (2010). CD-HIT Suite: a web server for clustering and comparing biological sequences. Bioinformatics.

[61] Radford A, Wu J, Child R, Luan D, Amodei D, Sutskever I (2019). Language models are unsupervised multitask learners. OpenAI blog.

[62] Devlin J, Chang M-W, Lee K, Toutanova K: Bert: pre-training of deep bidirectional transformers for language understanding. Proceedings of the 2019 Conference of the North American Chapter of the Association for Computational Linguistics. 2019: 4171–4186.

[63] Vu MH, Akbar R, Robert PA, Swiatczak B, Sandve GK, Greiff V, Haug DTT (2023). Linguistically inspired roadmap for building biologically reliable protein language models. Nat Mach Intell.

[64] Elnaggar A, Heinzinger M, Dallago C, Rihawi G, Wang Y, Jones L, Gibbs T, Feher T, Angerer C, Steinegger M (2020). ProtTrans: towards cracking the language of life’s code through self-supervised deep learning and high performance computing. IEEE Trans Pattern Anal Mach Intell.

[65] Li H, Liu B (2023). BioSeq-Diabolo: biological sequence similarity analysis using Diabolo. PLOS Comput Biol.

[66] Chung J, Gulcehre C, Cho K, Bengio Y: Empirical evaluation of gated recurrent neural networks on sequence modeling. Twenty-eighth Conference on Neural Information Processing Systems (Workshops). 2014: 1–9.

[67] Sutskever I, Vinyals O, Le QV: Sequence to sequence learning with neural networks. Twenty-eighth Conference on Neural Information Processing Systems. 2014: 1–9.

[68] Shannon CE (1948). A mathematical theory of communication. Bell syst tech j.

[69] Quinlan JR (1986). Induction of decision trees. Mach learn.

[70] Velickovic P, Cucurull G, Casanova A, Romero A, Lio P, Bengio Y (2017). Graph attention networks. Stat.

[71] Defferrard M, Bresson X, Vandergheynst P: Convolutional neural networks on graphs with fast localized spectral filtering. Advances in Neural Information Processing Systems. 2016: 3844–3852.

[72] He T, Hu J, Song Y, Guo J, Yi Z (2020). Multi-task learning for the segmentation of organs at risk with label dependence. Med Image Anal.

[73] Wang Y, Zhai Y, Ding Y, Zou Q: SBSM-Pro: support bio-sequence machine for proteins. arXiv preprint arXiv:230810275 2023.

[74] Dao FY, Liu ML, Su W, Lv H, Zhang ZY, Lin H, Liu L (2023). AcrPred: a hybrid optimization with enumerated machine learning algorithm to predict Anti-CRISPR proteins. Int j biol macromol.

[75] Zou X, Ren L, Cai P, Zhang Y, Ding H, Deng K, Yu X, Lin H, Huang C (2023). Accurately identifying hemagglutinin using sequence information and machine learning methods. Front med.

[76] Zhu W, Yuan SS, Li J, Huang CB, Lin H, Liao B (2023). A first computational frame for recognizing heparin-binding protein. Diagnostics.

[77] Ao C, Ye X, Sakurai T, Zou Q, Yu L (2023). m5U-SVM: identification of RNA 5-methyluridine modification sites based on multi-view features of physicochemical features and distributed representation. Bmc Biol.

[78] Tang FR, Chao JN, Wei YM, Yang FL, Zhai YX, Xu L, Zou Q (2022). HAlign 3: fast multiple alignment of ultra-large numbers of similar DNA/RNA sequences. Mol Biol Evol.

[79] Zou Q, Hu Q, Guo M, Wang G (2015). HAlign: fast multiple similar DNA/RNA sequence alignment based on the centre star strategy. Bioinformatics.

[80] Steinegger M, Meier M, Mirdita M, Vohringer H, Haunsberger SJ, Soding J (2019). HH-suite3 for fast remote homology detection and deep protein annotation. BMC Bioinformatics.

[81] Avanti S, Peyton GA, Kundaje: Learning important features through propagating activation differences. Proceedings of the 34th International Conference on Machine Learning. 2017: 3145–3153.

[82] Schwarzenberg R, Hübner M, Harbecke D, Alt C, Hennig L: Layerwise relevance visualization in convolutional text graph classifiers. Proceedings of the Thirteenth Workshop on Graph-Based Methods for Natural Language Processing. 2019: 58–62.

[83] Sheehy AM, Gaddis NC, Choi JD, Malim MH (2002). Isolation of a human gene that inhibits HIV-1 infection and is suppressed by the viral Vif protein. Nature.

[84] Mercenne G, Bernacchi S, Richer D, Bec G, Henriet S, Paillart JC, Marquet R (2010). HIV-1 Vif binds to APOBEC3G mRNA and inhibits its translation. Nucleic Acids Res.

[85] Bennett RP, Salter JD, Smith HC (2018). A new class of antiretroviral enabling innate immunity by protecting APOBEC3 from HIV Vif-dependent degradation. Trends Mol Med.

[86] Rose KM, Marin M, Kozak SL, Kabat D (2004). The viral infectivity factor (Vif) of HIV-1 unveiled. Trends Mol Med.

[87] Yu L, Yang K, He X, Li M, Gao L, Zha Y (2023). Repositioning linifanib as a potent anti-necroptosis agent for sepsis. Cell Death Discov.

[88] Ito F, Alvarez-Cabrera AL, Liu S, Yang H, Shiriaeva A, Zhou ZH, Chen XS (2023). Structural basis for HIV-1 antagonism of host APOBEC3G via Cullin E3 ligase. Sci Adv.

[89] Burley SK, Bhikadiya C, Bi C, Bittrich S, Chao H, Chen L, Craig PA, Crichlow GV, Dalenberg K, Duarte JM (2023). RCSB Protein Data Bank (RCSB.org): delivery of experimentally-determined PDB structures alongside one million computed structure models of proteins from artificial intelligence/machine learning. Nucleic Acids Res.

[90] Reingewertz TH, Benyamini H, Lebendiker M, Shalev DE, Friedler A (2009). The C-terminal domain of the HIV-1 Vif protein is natively unfolded in its unbound state. Protein Eng Des Sel.

