# Supplementary material for: DisoFLAG: accurate prediction of protein intrinsic disorder and its functions using graph-based interaction protein language model
Source: BMC Biol. 2024 Jan 2;22:3. doi: 10.1186/s12915-023-01803-y (PMC10762911; doi:10.1186/s12915-023-01803-y)
Supplement: Supplementary file 1 — Additional file 1: Fig. S1. Visualization of the IG matrix. Table S1. The ontology term and its sub-terms for each disordered functional class. Table S2. The statistical information of the datasets. Table S3. The number of trainable variables and hyper-parameters of DisoFLAG. Table S4. The definition of evaluation metrics. Table S5. The performance ranking of DisoFLAG using different features. Table S6. The statistical significance of differences (p-value) in predictive performance by different methods on the DP93 test dataset. Table S7. Performance comparisons of DisoFLAG and other predictors on the DP94 independent test dataset. Table S8. The statistical significance of differences (p-value) in predictive performance by different methods on the DP94 test dataset. Table S9. Per-protein performance of different disordered function predictors on the DP93 test dataset. Table S10. Per-protein performance of different disordered function predictors on the DP94 test dataset. Table S11. Performance metrics for Disorder-Binding prediction on the CAID2 test dataset. Table S12. Performance metrics for Disorder-Linker prediction on the CAID2 test dataset. Table S13. Performance metrics for disorder prediction on the CAID2 Disorder-NOX and Disorder-PDB test datasets. Table S14. Per-protein performance of different disorder predictors on the CAID2 Disorder-NOX and Disorder-PDB test datasets. [file 12915_2023_1803_MOESM1_ESM.pdf]

Figure S1. Visualization of the IG matrix of six disordered functions computed on the training dataset.

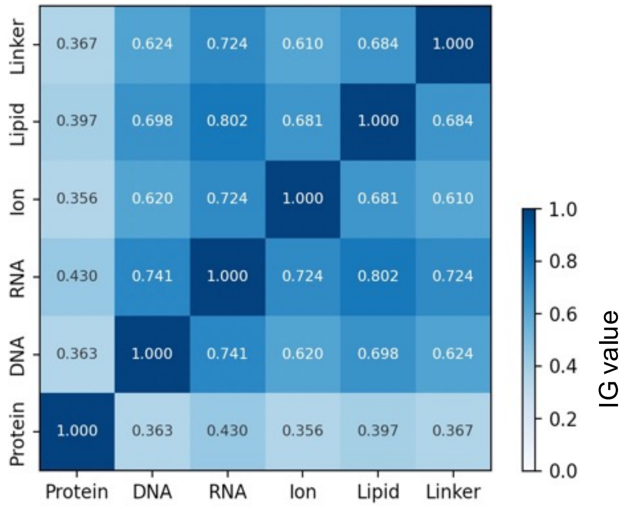

Table S1. The ontology term and its sub-terms for each disordered functional class from the DisProt database.

Functional class	Sub-term names
Protein binding	Protein binding<br>P53 binding<br>TBP-class protein binding<br>Beta-catenin binding<br>Calmodulin binding<br>Transcription coactivator binding<br>Growth factor binding<br>Histone binding<br>SH3 domain binding<br>Ubiquitin protein ligase binding<br>Platelet-derived growth factor receptor binding<br>Protein kinase binding<br>MHC class I protein binding<br>MDM2/MDM4 family protein binding<br>14-3-3 protein binding<br>RNA polymerase binding<br>Importin-alpha family protein binding
DNA binding	DNA binding, bending<br>DNA binding<br>Single-stranded DNA binding
RNA binding	RNA binding<br>Single-stranded RNA binding<br>G-quadruplex RNA binding<br>Regulatory region RNA binding<br>RNA stem-loop binding<br>rRNA binding<br>mRNA binding<br>tRNA binding
Ion binding	Ion binding<br>Iron ion binding<br>Zinc ion binding<br>Copper ion binding<br>Potassium ion binding<br>Metal ion binding<br>Calcium ion binding
Lipid binding	Lipid binding
Flexible linker	Flexible linker/spacer

Table S2. The statistical information of the datasets.

**Dataset**	**Number of proteins**	**Number of residues**					
	**All**	**Protein binding**	**DNA binding**	**RNA binding**	**Ion binding**	**Lipid binding**	**Flexible linker**
Training	589	36399	4579	1503	5298	2668	5249
Validation	148	9798	303	886	446	657	1212
DP93	188	9606	539	1430	667	1530	2029
DP94	98	6283	195	250	711	602	3172

Table S3. The number of trainable variables and hyper-parameters of DisoFLAG.

Module	Layer	Parameters	Explanation	Number/ value
GiPLM	Bi-GRU layer	$\mathbf{r}_i$	Residue-wise PLM embedding	1×1024
		$\mathbf{P}$	Contextual semantic encodings for sequence	L×1024
	Attention-based GRU layer	$\mathbf{p}_i$	Contextual semantic encodings for i-th residue	1×2048
		$\mathbf{W}_a$	Weight variables for calculating the attention score	1×2048
		s_{ij}	Attention score between i-th and j-th residues	1
		α_{ij}	Attention weight between i-th and j-th residues	1
		$\mathbf{c}_i$	Contextual vectors calculated for i-th residue	1×2048
		$\mathbf{h}_i$	The output hidden representations for i-th residue	1×2048
	Feature mapping layer n=[1,2⋯,6]	$\mathbf{W}^{(n)}$	The weight variables of the fully connected layer	2048×1024
		$\mathbf{b}^{(n)}$	The bias variables of the fully connected layer	1×1024
		$\mathbf{X}_i^{(n)}$	The output functional semantic representations for i-th residue	1×1024
	Graph-based interaction unit	$\mathbf{A}$	Weighted adjacency matrix of the edges	6× 6
		$\mathbf{X}$	Functional semantic representation vectors of the nodes	6×1024
	GCN layer n=[1,2⋯,6]	A_{ij}	The IG value between i-th and j-th functions	
		$\mathbf{X}_i$	The semantic representations of i-th function	6×1024
		$\mathbf{W}^{(n)\prime}$	The weight variables of the GCN layer	1024×128
		$\mathbf{b}^{(n)\prime}$	The bias variables of the GCN layer	1 ×128
		$\mathbf{Y}_i^{(n)}$	The aggregated semantic features for n-th function of i-th residue	1 ×128
	Max pooling	Kernel size	The shape of kernel in max pooling layer	6 ×1
		Stride	The shape of stride in max pooling layer	6 ×1
		$\mathbf{Y}_i^{IDR}$	The disordered semantic features for i-th residue	1 ×128
		F	The dimension of node features	128
	Fully connected layers n=[1,2⋯,7]	$\mathbf{W}^{(n)\prime\prime}$	The weight variables of the fully connected layer	128× 1
		$\mathbf{b}^{(n)\prime\prime}$	The bias variables of the fully connected layer	1×1
		Activation	The activation function of each layer	Sigmoid
Training	L		Maximum sequence length	128
	d_r		Dropout rate in each layer	0.3
	epoch		Number of times the training dataset used to train the model.	50
	Batch size		Number of samples in each iteration of updates to the model	16
	learning rate		Model learning rate	0.0005

Table S4. The definition of evaluation metrics.

Metric	Definition
TPR (true positive rate)	$\mathrm{TPR} = \frac{\mathrm{TP}}{\mathrm{TP} + \mathrm{FN}}$
FPR (false positive rate)	$\mathrm{FPR} = \frac{\mathrm{FP}}{\mathrm{FP} + \mathrm{TN}}$
TNR (true negative rate)	$\mathrm{TNR} = \frac{\mathrm{TN}}{\mathrm{TN} + \mathrm{FP}}$
Precision	$\mathrm{Precision} = \frac{\mathrm{TP}}{\mathrm{TP} + \mathrm{FP}}$
Recall	$\mathrm{Recall} = \frac{\mathrm{TP}}{\mathrm{TP} + \mathrm{FN}}$
F_{max}	$\mathrm{F}_{\mathrm{max}} = max\ (\frac{2 \times \mathrm{Precision} \times \mathrm{Recall}}{\mathrm{recision} + \mathrm{Recall}})$
MCC (Matthews correlation coefficient)	$\mathrm{F}_{\mathrm{max}} = \frac{\mathrm{TP} \times \mathrm{TN} - \mathrm{FP} \times \mathrm{FN}}{\sqrt{(\mathrm{TP} + \mathrm{FP}) \times (\mathrm{TP} + \mathrm{FN}) \times (\mathrm{TN} + \mathrm{FP}) \times (\mathrm{TN} + \mathrm{FN})}}$
BACC (balanced accuracy)	$\mathrm{BACC} = max\ (\frac{\mathrm{TPR} + \mathrm{TNR}}{2})$
TP (true positive)	Number of correctly predicted positive samples
TN (true negative)	Number of correctly predicted negative samples
FP (false positive)	Number of negative samples predicted as positives
FN (false negative)	Number of positive samples predicted as negatives

Table S5. The performance ranking of DisoFLAG using different features.

Prediction	ProtT5	ProtBERT	PSSM	One-hot
Disorder	1	2	3	4
Protein binding	1	2	3	4
DNA binding	1	2	3	4
RNA binding	1	3	2	4
Ion binding	1	2	3	4
Lipid binding	1	2	3	4
Flexible linker	1	3	2	4
Overall	7	16	19	28

Table S6. The statistical significance of differences (*p*-value) in predictive performance by different methods on the DP93 test dataset. The *p*-values are calculated by resampling half of the test dataset 20 times and using the two-sided paired t-test for each pair of the disordered function predictors. The upper triangle compares AUC value, and the lower triangle compares F_{max} value. The *p*-value > 0.05 is highlighted in bold.

Prediction	Methods	DisoFLAG	fIDPnn	Deep DISOBind	DisoRDP bind	ANCH OR-2	MoRFchib i-Light	SPOT-MoRF	MoRFchib i-Web
Disorder protein binding	DisoFLAG	\	0.00070	0.00008	0.00000	0.00000	0.00000	0.00000	0.00000
	fIDPnn	0.02518	\	0.01789	0.00001	0.00000	0.00000	0.00000	0.00000
	DeepDISOBind	0.00932	0.04625	\	0.00077	0.00000	0.00000	0.00000	0.00000
	DisoRDPbind	0.00196	0.00068	0.00002	\	0.00002	0.00000	0.00000	0.00000
	ANCHOR-2	0.00000	0.00005	0.00035	0.00057	\	0.00237	0.00257	0.00000
	MoRFchibi-Light	0.00000	0.00000	0.00000	0.00000	0.00008	\	0.16224	0.00014
	SPOT-MoRF	0.00000	0.00000	0.00000	0.00000	0.00000	0.02881	\	0.00019
	MoRFchibi-Web	0.00000	0.00000	0.00000	0.00000	0.00000	0.00010	0.00132	\

Prediction	Methods	DisoFLAG	fIDPnn	DeepDISO Bind	DisoRDP bind
Disorder DNA binding	DisoFLAG	\	0.00072	0.00000	0.00000
	fIDPnn	0.01938	\	0.00010	0.00015
	DeepDISOBind	0.00000	0.00000	\	**0.05898**
	DisoRDPbind	0.01169	0.01260	0.00001	\

Prediction	Methods	DisoFLAG	fIDPnn	DeepDISO Bind	DisoRDP bind
Disorder RNA binding	DisoFLAG	\	0.00027	0.00000	0.00000
	fIDPnn	0.00712	\	**0.07384**	0.00000
	DeepDISOBind	0.00001	0.00000	\	0.00000
	DisoRDPbind	0.00000	0.00000	0.00000	\

Prediction	Methods	DisoFLAG
Disorder Ion binding	DisoFLAG	\

Prediction	Methods	DisoFLAG	DisoLipP red
Disorder lipid binding	DisoFLAG	\	0.00000
	DisoLipPred	0.00000	\

Prediction	Methods	DisoFLAG	fIDPnn	TransDFL	DFLpred
Flexible linker	DisoFLAG	\	0.00000	0.00000	0.00000
	fIDPnn	0.00000	\	0.00000	0.00000
	TransDFL	0.00000	0.00000	\	0.00000
	DFLpred	0.00000	**0.15898**	0.00000	\

Table S7. Performance comparisons of DisoFLAG and other predictors on the DP94 independent test dataset.

Prediction	Method*	AUC	AUPR	F_{max}	MCC	BACC
Protein binding	fIDPnn†	0.857	0.311	0.372	0.338	0.786
	DisoRDPbind†	0.851	0.225	0.313	0.289	0.785
	DisoFLAG	0.846	0.285	0.357	0.327	0.784
	DeepDISOBind†	0.818	0.149	0.274	0.270	0.774
	ANCHOR-2††	0.807	0.157	0.272	0.274	0.767
	MoRFchibi-Light†	0.795	0.157	0.236	0.217	0.724
	MoRFchibi-Web†	0.765	0.157	0.233	0.190	0.696
	SPOT-MoRF††	0.650	0.165	0.263	0.191	0.645
DNA binding	fIDPnn†	0.895	0.010	0.032	0.073	0.843
	DisoRDPbind†	0.724	0.003	0.009	0.033	0.707
	DisoFLAG	0.716	0.004	0.016	0.035	0.685
	DeepDISOBind†	0.621	0.002	0.007	0.021	0.618
RNA binding	DeepDISOBind†	0.950	0.078	0.212	0.244	0.891
	fIDPnn†	0.889	0.016	0.052	0.115	0.833
	DisoRDPbind†	0.875	0.019	0.055	0.118	0.807
	DisoFLAG	0.833	0.006	0.016	0.064	0.801
Ion binding	DisoFLAG	0.930	0.706	0.817	0.829	0.849
Lipid binding	DisoFLAG	0.800	0.021	0.067	0.085	0.701
	DisoLipPred†	0.799	0.014	0.031	0.072	0.749
Flexible linker	DisoFLAG	0.762	0.110	0.184	0.167	0.685
	fIDPnn†	0.725	0.050	0.097	0.117	0.686
	TransDFL††	0.530	0.071	0.067	0.027	0.541
	DFLpred†	0.466	0.024	0.049	0.014	0.508

* The evaluation results of the comparative methods were calculated based on the results obtained by running their respective web servers† and standalone packages††. Predictors in each prediction are sorted by their AUC value.

Table S8. The statistical significance of differences (*p*-value) in predictive performance by different methods on the DP94 test dataset. The *p*-values are calculated by resampling half of the test dataset 20 times and using the two-sided paired t-test for each pair of the disordered function predictors. The upper triangle compares AUC value, and the lower triangle compares F_{max} value. The *p*-value > 0.05 is highlighted in bold.

Prediction	Methods	DisoFLAG	fIDPnn	Deep DISOBind	DisoRDP bind	ANCH OR-2	MoRFchib i-Light	SPOT-MoRF	MoRFchib i-Web
Disorder protein binding	DisoFLAG	\	0.00997	0.00262	0.0429	0.00344	0.00015	0.00000	0.00263
	fIDPnn	0.00275	\	0.00005	0.03848	0.00003	0.00001	0.00000	0.00000
	DeepDISOBind	0.00015	0.00025	\	0.0004	**0.15342**	0.00005	0.00000	0.00001
	DisoRDPbind	0.00025	0.00599	0.00249	\	0.00082	0.00001	0.00000	0.00000
	ANCHOR-2	0.00016	0.00022	0.00708	0.00173	\	0.00542	0.00000	0.00004
	MoRFchibi-Light	0.00000	0.00000	0.00136	0.00002	0.00292	\	0.00000	0.00172
	SPOT-MoRF	0.00000	0.00000	**0.07088**	0.00084	**0.07642**	0.00861	\	0.00000
	MoRFchibi-Web	0.00000	0.00000	0.00021	0.00025	0.00815	0.00766	0.00367	\

Prediction	Methods	DisoFLAG	fIDPnn	DeepDISO Bind	DisoRDP bind
Disorder DNA binding	DisoFLAG	\	0.00000	0.00480	0.00000
	fIDPnn	0.00019	\	0.00000	0.00000
	DeepDISOBind	0.00941	0.00012	\	0.00000
	DisoRDPbind	0.00006	0.00004	0.0073	\

Prediction	Methods	DisoFLAG	fIDPnn	DeepDISO Bind	DisoRDP bind
Disorder RNA binding	DisoFLAG	\	0.00000	0.00000	0.00000
	fIDPnn	0.00000	\	0.00000	0.00000
	DeepDISOBind	0.00000	0.00000	\	0.00000
	DisoRDPbind	0.00000	**0.10077**	0.00000	\

Prediction	Methods	DisoFLAG
Disorder Ion binding	DisoFLAG	\

Prediction	Methods	DisoFLAG	DisoLipP red
Disorder lipid binding	DisoFLAG	\	0.01707
	DisoLipPred	0.00000	\

Prediction	Methods	DisoFLAG	fIDPnn	TransDFL	DFLpred
Flexible linker	DisoFLAG	\	0.00001	0.00000	0.00000
	fIDPnn	0.00000	\	0.00000	0.00000
	TransDFL	0.00000	0.00000	\	0.00000
	DFLpred	0.00000	0.00000	0.00000	\

Table S9. Per-protein performance of different disordered function predictors on the DP93 test dataset. Metrics are averaged over the protein sequence.

Prediction	Method*	F_{max}	MCC	BACC	TPR	TNR	PPV	C
Protein binding	DisoFLAG	0.263	0.164	0.546	0.381	0.712	0.272	1.00
	SPOT-MoRF	0.247	0.106	0.486	0.492	0.480	0.202	0.67
	fIDPnn	0.235	0.116	0.507	0.336	0.677	0.238	1.00
	MoRFchibi-Light	0.226	0.076	0.471	0.389	0.554	0.201	1.00
	MoRFchibi-Web	0.223	0.075	0.460	0.397	0.522	0.197	1.00
	DisoRDPbind	0.218	0.130	0.518	0.344	0.692	0.216	1.00
	ANCHOR-2	0.193	0.104	0.512	0.304	0.721	0.208	1.00
	DeepDISOBind	0.126	0.060	0.505	0.168	0.842	0.145	1.00
DNA binding	DisoFLAG	0.023	0.022	0.498	0.026	0.970	0.026	1.00
	DeepDISOBind	0.020	0.009	0.365	0.033	0.697	0.022	1.00
	fIDPnn	0.006	0.006	0.498	0.005	0.991	0.011	1.00
	DisoRDPbind	0.005	0.005	0.497	0.005	0.990	0.005	1.00
RNA binding	fIDPnn	0.049	0.013	0.376	0.050	0.703	0.050	1.00
	DeepDISOBind	0.031	0.002	0.482	0.029	0.936	0.039	1.00
	DisoRDPbind	0.025	0.000	0.390	0.022	0.758	0.039	1.00
	DisoFLAG	0.022	0.015	0.460	0.019	0.902	0.054	1.00
Ion binding	DisoFLAG	0.019	0.007	0.227	0.047	0.407	0.015	1.00
Lipid binding	DisoLipPred	0.023	0.010	0.440	0.022	0.858	0.032	1.00
	DisoFLAG	0.013	0.003	0.489	0.011	0.968	0.020	1.00
Flexible linker	DisoFLAG	0.103	0.102	0.540	0.101	0.979	0.132	1.00
	TransDFL	0.076	0.078	0.535	0.070	0.999	0.096	1.00
	fIDPnn	0.066	0.045	0.424	0.080	0.769	0.076	1.00
	DFLpred	0.041	0.027	0.490	0.054	0.925	0.054	1.00

* Methods are sorted by F_{max} values. MCC, Matthews correlation coefficient; BACC, balanced accuracy; TPR, true positive rate; TNR, true negative rate; PPV, positive predictive value, *i.e.*, precision; C, coverage of predictions.

Table S10. Per-protein performance of different disordered function predictors on the DP94 test dataset. Metrics are averaged over the protein sequence.

Prediction	Method*	F_{max}	MCC	BACC	TPR	TNR	PPV	C
Protein binding	SPOT-MoRF	0.219	0.079	0.423	0.534	0.311	0.169	0.67
	DisoFLAG	0.194	0.147	0.555	0.229	0.881	0.215	1.00
	ANCHOR-2	0.188	0.126	0.546	0.294	0.798	0.178	1.00
	MoRFchibi-Light	0.179	0.086	0.517	0.305	0.729	0.167	1.00
	DeepDISOBind	0.178	0.125	0.539	0.259	0.819	0.204	1.00
	flDPnn	0.169	0.105	0.543	0.228	0.857	0.198	1.00
	DisoRDPbind	0.160	0.119	0.547	0.188	0.905	0.211	1.00
	MoRFchibi-Web	0.154	0.064	0.507	0.207	0.807	0.166	1.00
DNA binding	flDPnn	0.007	0.006	0.471	0.007	0.936	0.012	1.00
	DisoRDPbind	0.006	0.005	0.401	0.017	0.786	0.004	1.00
	DisoFLAG	0.005	0.004	0.469	0.013	0.924	0.003	1.00
	DeepDISOBind	0.004	0.004	0.456	0.009	0.903	0.003	1.00
RNA binding	flDPnn	0.017	0.017	0.452	0.021	0.883	0.021	1.00
	DeepDISOBind	0.013	0.014	0.502	0.010	0.993	0.020	1.00
	DisoFLAG	0.011	0.007	0.360	0.021	0.698	0.008	1.00
	DisoRDPbind	0.009	0.009	0.485	0.014	0.957	0.007	1.00
Ion binding	DisoFLAG	0.010	0.007	0.503	0.010	0.996	0.010	1.00
Lipid binding	DisoLipPred	0.016	0.014	0.409	0.025	0.793	0.017	1.00
	DisoFLAG	0.010	0.008	0.494	0.009	0.980	0.010	1.00
Flexible linker	DisoFLAG	0.101	0.101	0.549	0.142	0.957	0.111	1.00
	flDPnn	0.071	0.065	0.393	0.260	0.527	0.050	1.00
	DFLpred	0.048	0.004	0.200	0.388	0.012	0.027	1.00
	TransDFL	0.026	0.028	0.509	0.017	0.999	0.051	1.00

* Methods are sorted by F_{max} values. MCC, Matthews correlation coefficient; BACC, balanced accuracy; TPR, true positive rate; TNR, true negative rate; PPV, positive predictive value, *i.e.*, precision; C, coverage of predictions.

Table S11. Performance metrics for Disorder-Binding prediction on the CAID2 test dataset.

Prediction	Method*	AUC	APS	F_{max}	MCC	BACC	C
Disorder-Binding	DisoFLAG-Protein	0.879	0.563	0.554	0.490	0.802	1.00
	DisoFLAG-Lipid	0.847	0.498	0.511	0.438	0.783	1.00
	DisoFLAG-Ion	0.840	0.426	0.495	0.417	0.759	1.00
	ENSHROUD-protein	0.753	0.252	0.360	0.288	0.720	1.00
	DisoFLAG-RNA	0.752	0.285	0.378	0.277	0.687	1.00
	MoRFchibi-web	0.751	0.284	0.355	0.263	0.695	1.00
	DeepDRPBind-protein	0.744	0.285	0.366	0.273	0.698	1.00
	MoRFchibi-light	0.740	0.272	0.341	0.238	0.677	1.00
	DeepDISOBind	0.733	0.217	0.351	0.279	0.712	1.00
	OPAL	0.724	0.284	0.353	0.248	0.673	1.00
	DRPBind-protein	0.721	0.249	0.340	0.257	0.696	1.00
	DisoRDPbind-protein	0.710	0.227	0.332	0.241	0.683	0.99
	ANCHOR2	0.699	0.198	0.325	0.226	0.671	1.00
	CLIP	0.694	0.252	0.344	0.236	0.659	1.00
	AlphaFold-binding	0.688	0.270	0.373	0.239	0.652	0.79
	DisoFLAG-DNA	0.601	0.175	0.251	0.107	0.576	1.00

* Methods are sorted by AUC values. C, coverage of predictions.

Table S12. Performance metrics for Disorder-Linker prediction on the CAID2 test dataset.

Prediction	Method*	AUC	APS	F_{max}	MCC	BACC	C
Disorder-Linker	DisoFLAG-Linker	0.800	0.197	0.258	0.241	0.736	1.00
	SPOT-Disorder2	0.782	0.153	0.292	0.276	0.749	0.78
	AlphaFold-rsa	0.770	0.103	0.224	0.251	0.774	0.93
	SETH-0	0.770	0.158	0.219	0.216	0.724	1.00
	SETH-1	0.762	0.133	0.228	0.206	0.712	1.00
	Dispredict3	0.744	0.148	0.275	0.243	0.703	1.00
	AUCpreD	0.734	0.117	0.212	0.190	0.695	1.00
	APOD	0.729	0.133	0.241	0.206	0.689	1.00
	PredIDR-short	0.728	0.119	0.203	0.151	0.667	1.00
	PredIDR-long	0.722	0.111	0.195	0.165	0.680	1.00
	PreDisorder	0.718	0.156	0.266	0.200	0.674	0.97
	flDPnn2	0.708	0.138	0.270	0.227	0.669	1.00
	flDPlr2	0.691	0.136	0.245	0.208	0.677	1.00

* Methods are sorted by AUC values. C, coverage of predictions.

Table S13. Performance metrics for disorder prediction on the CAID2 Disorder-NOX and Disorder-PDB test datasets.

Dataset	Method*	AUC	APS	F_{max}	MCC	BACC	C
Disorder-NOX	Dispredict3	0.838	0.581	0.548	0.429	0.765	1.00
	DisoFLAG	0.836	0.560	0.548	0.428	0.753	1.00
	flDPnn	0.833	0.586	0.546	0.424	0.760	1.00
	DisoPred	0.821	0.504	0.549	0.433	0.766	0.92
	flDPlr2	0.821	0.562	0.532	0.405	0.749	1.00
	IDP-Fusion	0.818	0.474	0.539	0.423	0.764	0.97
	ESpritz-D	0.802	0.470	0.520	0.399	0.750	1.00
	DeepIDP-2L	0.800	0.460	0.513	0.386	0.743	1.00
	disomine	0.797	0.459	0.515	0.391	0.746	1.00
	rawMSA	0.783	0.468	0.489	0.357	0.725	1.00
	SPOT-Disorder2	0.780	0.558	0.632	0.451	0.747	0.83
Disorder-PDB	SPOT-Disorder2	0.949	0.928	0.860	0.795	0.891	0.82
	AlphaFold-rsa	0.944	0.916	0.849	0.788	0.891	0.86
	PredIDR-long	0.934	0.870	0.800	0.723	0.867	1.00
	IDP-Fusion	0.933	0.878	0.822	0.756	0.876	0.98
	SPOT-Disorder	0.931	0.889	0.823	0.758	0.875	1.00
	SETH-0	0.930	0.893	0.830	0.772	0.877	1.00
	PredIDR-short	0.927	0.859	0.790	0.709	0.859	1.00
	AUCpreD	0.924	0.867	0.802	0.726	0.860	0.99
	metapredict	0.923	0.877	0.819	0.758	0.867	1.00
	DeepIDP-2L	0.922	0.858	0.794	0.712	0.862	1.00
	DisoFLAG	0.920	0.850	0.764	0.669	0.847	1.00
	DisoPred	0.919	0.859	0.784	0.706	0.855	0.95
	SPOT-Disorder-Single	0.917	0.870	0.791	0.716	0.854	1.00

* Methods are sorted by AUC values. C, coverage of predictions.

Table S14. Per-protein performance of different disorder predictors on the CAID2 Disorder-NOX and Disorder-PDB test datasets. Metrics are averaged over the protein sequence.

Prediction	Method*	F_{max}	MCC	BACC	TPR	TNR	PPV	C
Disorder-NOX	DisoFLAG	0.524	0.294	0.665	0.706	0.624	0.515	1.00
	SPOT-Disorder2	0.353	0.171	0.397	0.471	0.323	0.339	0.83
	Dispredict3	0.307	0.130	0.377	0.451	0.3030	0.283	1.00
	rawMSA	0.305	0.152	0.389	0.465	0.312	0.287	1.00
	IDP-Fusion	0.302	0.145	0.374	0.418	0.331	0.301	0.97
	flDPlr2	0.301	0.140	0.378	0.444	0.311	0.288	1.00
	flDPnn	0.297	0.134	0.373	0.408	0.337	0.289	1.00
	ESpritz-D	0.293	0.123	0.364	0.415	0.313	0.282	1.00
	DeepIDP-2L	0.291	0.143	0.370	0.403	0.337	0.300	1.00
	DisoPred	0.289	0.157	0.372	0.383	0.362	0.297	0.92
	disomine	0.288	0.119	0.358	0.402	0.314	0.282	1.00
Disorder-PDB	SPOT-Disorder2	0.697	0.395	0.682	0.722	0.642	0.737	0.82
	AlphaFold-rsa	0.690	0.425	0.705	0.744	0.665	0.706	0.86
	SETH-0	0.671	0.413	0.681	0.678	0.684	0.746	1.00
	PredIDR-short	0.661	0.407	0.682	0.704	0.660	0.720	1.00
	PredIDR-long	0.652	0.404	0.675	0.682	0.667	0.726	1.00
	AUCpreD	0.648	0.366	0.679	0.721	0.638	0.673	0.99
	SPOT-Disorder	0.647	0.346	0.658	0.690	0.626	0.686	1.00
	IDP-Fusion	0.641	0.346	0.661	0.692	0.631	0.675	0.98
	DisoFLAG	0.630	0.308	0.658	0.756	0.560	0.637	1.00
	DeepIDP-2L	0.627	0.308	0.643	0.705	0.582	0.650	1.00
	metapredict	0.619	0.355	0.655	0.636	0.674	0.688	1.00
	SPOT-Disorder-Single	0.607	0.317	0.650	0.665	0.635	0.643	1.00
	DisoPred	0.572	0.284	0.626	0.618	0.634	0.631	0.95

* Methods are sorted by F_{max} values. C, coverage of predictions.
