# Supplementary figures and images for: DisoFLAG: accurate prediction of protein intrinsic disorder and its functions using graph-based interaction protein language model
Source: BMC Biol. 2024 Jan 2;22:3. doi: 10.1186/s12915-023-01803-y (PMC10762911; doi:10.1186/s12915-023-01803-y)

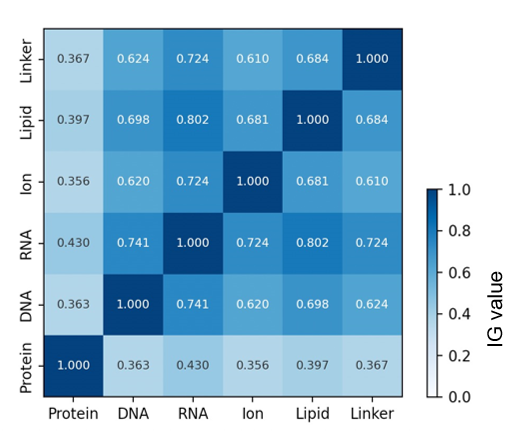

Supplement: Supplementary file 2 — Additional file 2. The data values for the figures. [file 12915_2023_1803_MOESM2_ESM.tif]
